# Resistance to chemical carcinogenesis induction via a dampened inflammatory response in naked mole-rats

**DOI:** 10.1038/s42003-022-03241-y

**Published:** 2022-03-30

**Authors:** Kaori Oka, Shusuke Fujioka, Yoshimi Kawamura, Yoshihiro Komohara, Takeshi Chujo, Koki Sekiguchi, Yuki Yamamura, Yuki Oiwa, Natsuko Omamiuda-Ishikawa, Shohei Komaki, Yoichi Sutoh, Satoko Sakurai, Kazuhito Tomizawa, Hidemasa Bono, Atsushi Shimizu, Kimi Araki, Takuya Yamamoto, Yasuhiro Yamada, Hiroyuki Oshiumi, Kyoko Miura

**Affiliations:** 1 Department of Aging and Longevity Research, Faculty of Life Sciences, Kumamoto University, Kumamoto, 860-0811 Japan; 2 Biomedical Animal Research Laboratory, Institute for Genetic Medicine, Hokkaido University, Sapporo, 060-0815 Japan; 3 Department of Cell Pathology, Faculty of Life Sciences, Kumamoto University, Kumamoto, 860-8556 Japan; 4 Department of Molecular Physiology, Faculty of Life Sciences, Kumamoto University, Kumamoto, 860-8556 Japan; 5 Division of Biomedical Information Analysis, Iwate Tohoku Medical Megabank Organization, Disaster Reconstruction Center, Iwate Medical University, Iwate, 028-3694 Japan; 6 Department of Life Science Frontiers, Center for iPS Cell Research and Application (CiRA), Kyoto University, Kyoto, 606-8507 Japan; 7 Center for Metabolic Regulation of Healthy Aging, Kumamoto University, Kumamoto, 860-8556 Japan; 8 Program of Biomedical Science, Graduate School of Integrated Sciences for Life, Hiroshima University, Hiroshima, 739-0046 Japan; 9 Division of Biomedical Information Analysis, Institute for Biomedical Sciences, Iwate Medical University, Iwate, 028-3694 Japan; 10 Institute of Resource Development and Analysis, Kumamoto University, Kumamoto, 860-0811 Japan; 11 Institute for the Advanced Study of Human Biology (WPI-ASHBi), Kyoto University, Kyoto, 606-8501 Japan; 12 Medical-risk Avoidance based on iPS Cells Team, RIKEN Center for Advanced Intelligence Project (AIP), Kyoto, 606-8507 Japan; 13 AMED-CREST, AMED, Tokyo, 100-0004 Japan; 14 Division of Stem Cell Pathology, Center for Experimental Medicine and Systems Biology, Institute of Medical Science, The University of Tokyo, Tokyo, 108-8639 Japan; 15 Department of Immunology, Faculty of Life Sciences, Kumamoto University, Kumamoto, 860-8556 Japan

**Keywords:** Cancer models, Cancer prevention

## Abstract

Naked mole-rats (NMRs) have a very low spontaneous carcinogenesis rate, which has prompted studies on the responsible mechanisms to provide clues for human cancer prevention. However, it remains unknown whether and how NMR tissues respond to experimental carcinogenesis induction. Here, we show that NMRs exhibit extraordinary resistance against potent chemical carcinogenesis induction through a dampened inflammatory response. Although carcinogenic insults damaged skin cells of both NMRs and mice, NMR skin showed markedly lower immune cell infiltration. NMRs harbour loss-of-function mutations in *RIPK3* and *MLKL* genes, which are essential for necroptosis, a type of necrotic cell death that activates strong inflammation. In mice, disruption of Ripk3 reduced immune cell infiltration and delayed carcinogenesis. Therefore, necroptosis deficiency may serve as a cancer resistance mechanism via attenuating the inflammatory response in NMRs. Our study sheds light on the importance of a dampened inflammatory response as a non-cell-autonomous cancer resistance mechanism in NMRs.

## Abstract (web summary)

Naked mole rats are found to be resistant to cancer development through dampened inflammatory response due to genetically determined impaired necroptosis, with essential necroptosis genes RIPK3 and MLKL containing mutations causing premature stop codons.

## Introduction

The naked mole-rat (NMR) is the longest-living rodent with a maximum lifespan of 37 years, despite being comparable size to the laboratory mice. Previous studies have reported that NMR is protected from age-associated decline in physiological functions and aging-related disorders^1,2^. In particular, spontaneous carcinogenesis has rarely been observed in over 2000 necropsies of captive NMR colonies^3,4^. This provides clear evidence of the cancer resistance properties of NMRs; however, to the best of our knowledge, it is the only evidence to date of their cancer resistance in vivo. Recently, however, some cases of NMR tumours including metastatic cancer have been reported^5–7^. Therefore, it is unclear how strongly NMR individuals are resistant to carcinogenesis. Moreover, there is no report regarding the tissue response of NMRs to experimental induction of carcinogenesis in vivo.

Intracellular mechanisms that may contribute to cancer resistance in NMRs have been proposed^8–10^; however, whether NMRs have strong cell-autonomous cancer resistance is currently debatable. Two reports have shown that NMR cells, unlike mouse cells, do not transform upon the introduction of HRasV12 and SV40 Large T antigen^9,11^. Conversely, another group recently reported that NMR cells do transform by the same treatment^12^. One limitation of previous studies is that the findings are based on the experimental transformation of cultured fibroblasts and their xenografts in immune-deficient mice.

In vivo carcinogenesis includes an initiation stage, in which DNA damage results in the generation of mutant cells. This is followed by changes in the tissue microenvironment around the mutant cells, which comprises surrounding immune cells and stromal cells. Microenvironmental changes regulate various environmental factors and promote carcinogenesis in a promotion stage^13,14^. In particular, tissue inflammation induces further genetic and epigenetic alterations of mutant cells and strongly promotes carcinogenesis in a non-cell-autonomous manner^15–18^. Therefore, previous studies on the cancer resistance of cultured NMR fibroblasts might not have paid enough attention to the physiological context of in vivo carcinogenesis and might overlook relevant cancer resistance mechanisms in NMR tissues.

Here, we show that NMR individuals exhibit extraordinary resistance to carcinogenesis induction by chemical carcinogens in vivo. Notably, NMR skin tissues showed an unusual dampened inflammatory response to carcinogenic insults. We found that NMRs harbour loss-of-function mutations in Receptor-interacting protein kinase 3 (*RIPK3*) and Mixed lineage kinase domain-like (*MLKL*) genes. These genes are the regulators of necroptosis, a type of strong inflammation-activating cell death associated with various inflammatory diseases^19^. Loss of necroptosis-inducing ability in NMRs may serve as a mechanism that attenuates inflammatory responses and suppresses carcinogenesis in vivo. This study highlights a dampened tissue inflammatory response as a non-cell-autonomous mechanism underlying carcinogenesis resistance in NMR individuals.

## Results

### Marked resistance to chemical carcinogenesis despite cellular damage in NMRs

**Fig. 1:**
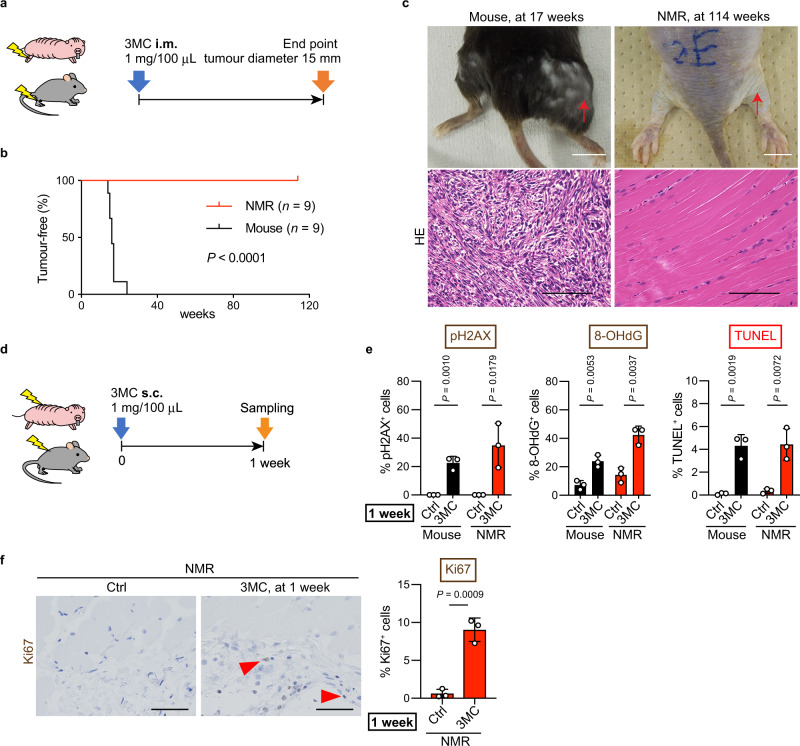
Naked mole-rats (NMRs) do not develop 3-methylcholanthrene (3MC) -induced tumours despite the induction of cellular damage. **a** Schematic diagram for carcinogenesis induction by intramuscular (i.m.) injection of 1 mg 3MC into the hind limb. **b** Kaplan–Meier curves of tumour-free mice and NMRs treated with 3MC. *n* = 9 animals per species. **c** Gross appearance and haematoxylin and eosin (HE) staining of a mouse tumour at 17 weeks and NMR muscle at 114 weeks after i.m. 3MC injection. Red arrows indicate injection sites. Scale bars: 1 cm (upper) and 50 μm (lower). **d** Schematic diagram for investigating short-term responses to 3MC after subcutaneous (s.c.) injection into the back skin. **e** Quantification of phospho-Histone H2A.X (pH2AX)-, 8-hydroxy-2′-deoxyguanosine (8-OHdG)-, and TUNEL-positive cells after s.c. 3MC-injection. **f** Immunohistochemical staining and quantification of Ki67-positive cells in NMR skin at 1 week after s.c. 3MC-injection. Red arrowheads show positive cells. Scale bar: 50 μm. For quantification of (**e** and **f**), data are presented as the mean ± SD of *n* = 3 animals. Log-rank test for (**b**). Unpaired *t*-test versus untreated control (Ctrl) for (**e** and **f**).

The in vivo responses of NMR tissues to carcinogenic insults were examined using two types of chemical carcinogens, and the effects were compared with those in mice. First, mice and NMRs received intramuscular injections of 3-methylcholanthrene (3MC), a carcinogen in various rodent species^20–22^ (Fig. 1a). After treatment, all mice developed fibrosarcomas within 24 weeks (9/9 tested animals). However, 3MC-treated NMRs did not develop tumours in a period of 114 weeks (0/9 tested animals; Fig. 1b, c and Supplementary Fig. 1a). Histopathological analysis (three animals) showed no obvious abnormalities (Fig. 1c). The remaining animals were kept alive, and no visible tumours were observed for 177 weeks. NMRs treated with the same amount of 3MC per g body weight as the mice did not form tumours after 49 weeks (0/9 tested animals; Supplementary Fig. 1b). Further, NMRs that received a subcutaneous injection of 3MC also did not develop visible tumours for 97 weeks, and no obvious histological abnormalities such as hyperplasia were detected (0/5 tested animals; Supplementary Fig. 1c–e). On the other hand, the mice developed severe skin ulcers and had to be euthanized within 10 weeks (Supplementary Fig. 1d).

To evaluate the early tissue responses to the carcinogen, 3MC was injected subcutaneously, and the effects were analysed after 1 week^23^ (Fig. 1d). NMR and mouse skin tissues showed increased phospho-histone H2A.X (pH2AX)-positive or 8-hydroxy-2′-deoxyguanosine (8-OHdG)-positive DNA-damaged cells in response to 3MC treatment, and TUNEL-positive dead cells were similarly increased (Fig. 1e and Supplementary Fig. 1f). Ki67-positive cells were increased in NMR skin tissues at 1 week and even at 97 weeks after 3MC treatment (Fig. 1f and Supplementary Fig. 1g). Thus, 3MC treatment increased DNA damage and activated tissue damage responses, such as cell death and proliferation, in NMR tissues, but had no tumorigenic effects.

**Fig. 2:**
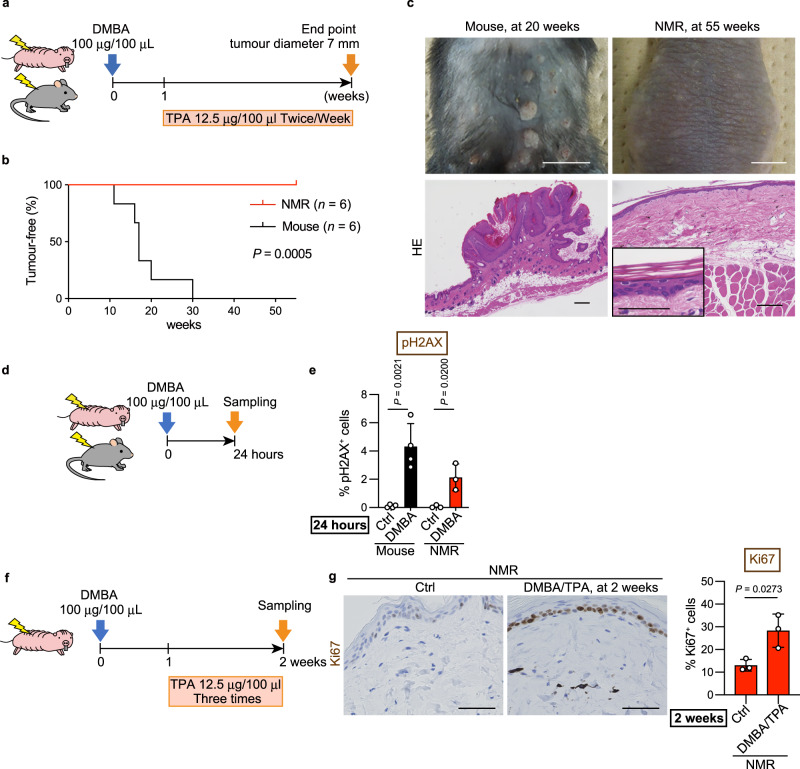
Naked mole-rats (NMRs) do not develop 7,12-dimethylbenz[a]anthracene (DMBA)/12-*O*-tetradecanoylphorbol-13-acetate (TPA)-induced tumours despite the induction of cellular damage. **a** Schematic diagram for carcinogenesis assessment by DMBA/TPA treatment on the back skin. **b** Kaplan–Meier curves of tumour-free mice and NMRs after DMBA/TPA treatment. *n* = 6 animals per species. **c** Gross appearance and haematoxylin and eosin (HE) staining of mouse papillomas at 20 weeks and NMR skin at 55 weeks after starting DMBA/TPA treatment. Scale bars: 1 cm (upper) and 100 μm (lower). Inset is a higher magnification of NMR skin (scale bar: 50 μm). **d** Schematic diagram for investigating short-term responses to DMBA treatment on the back skin. **e** Quantification of phospho-Histone H2A.X (pH2AX)-positive cells at 24 h after DMBA treatment. **f** Schematic diagram for investigating short-term responses to DMBA/TPA treatment on the back skin. **g** Immunohistochemical staining and quantification of Ki67-positive cells in NMR skin at 2 weeks after starting DMBA/TPA treatment. Scale bar: 50 μm. For quantification of (**e** and **g**), data are presented as the mean ± SD of *n* = 4 (for mouse) or *n* = 3 (for NMR) animals. Log-rank test for (**b**). Unpaired *t*-test versus untreated control (Ctrl) for (**e** and **g**).

Next, other carcinogens, namely, 7,12-dimethylbenz[a]anthracene (DMBA) and 12-*O*-tetradecanoylphorbol-13-acetate (TPA)^24^, were administered to the back skin of mice and NMRs (Fig. 2a). All mice developed multiple papillomas within 30 weeks (6/6 tested animals; Fig. 2b, c). On the other hand, NMRs did not develop any visible tumours at 55 weeks, and histopathological analysis of skin biopsies showed no obvious abnormalities (0/6 tested animals; Fig. 2b, c). These NMRs continued to receive TPA after biopsy and has not developed tumours at 116 weeks.

We then evaluated the tissue responses to DMBA/TPA at earlier stages. Similar to the effect of 3MC, DMBA treatment for 24 h significantly increased the number of pH2AX-positive DNA-damaged cells in both mouse and NMR skin (Fig. 2d, e and Supplementary Fig. 2a). Ki67-positive cells were significantly increased in NMR skin at 2 weeks and even at 55 weeks after DMBA/TPA treatment (Fig. 2f, g and Supplementary Fig. 2b). Taken together, these results demonstrate that treatment with carcinogenic agents increased DNA damage and cellular responses such as cell death and proliferation in NMR tissues. Despite this increasing tissue damage, NMR individuals showed marked resistance against two types of chemical carcinogenesis induction.

### Dampened tissue inflammatory responses after carcinogenic insults in NMRs

**Fig. 3:**
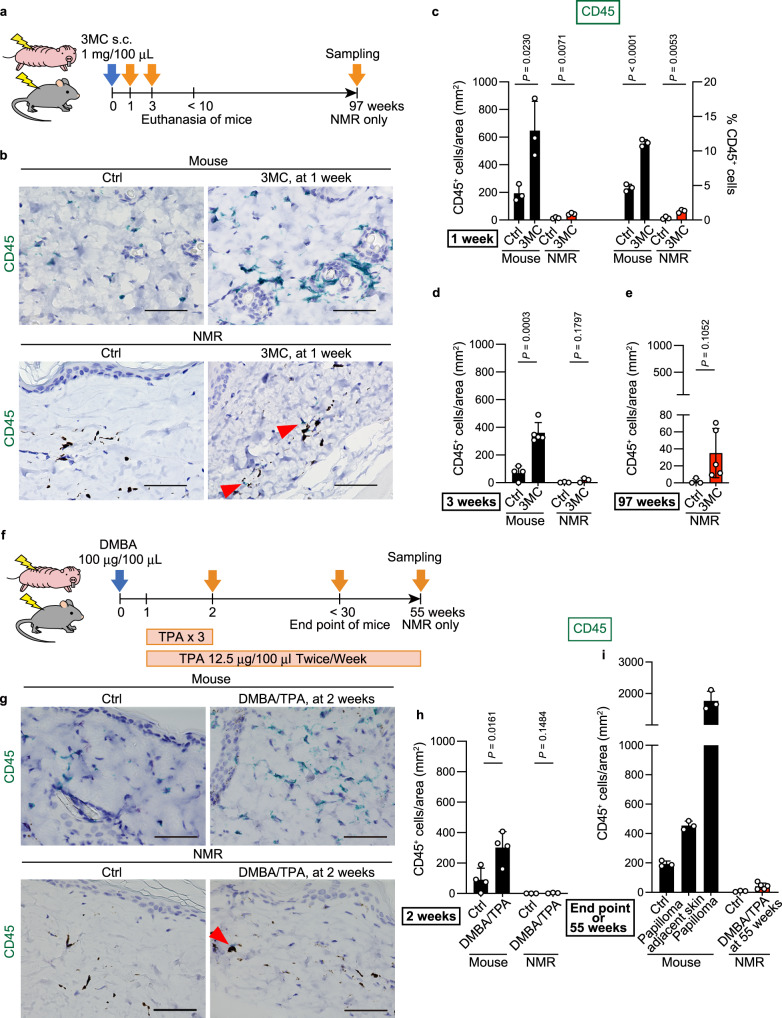
Attenuated infiltration of inflammatory immune cells in naked mole-rat (NMR) tissue upon administration of carcinogens. **a** Schematic diagram for investigating immune cell infiltration into the skin after a subcutaneous (s.c.) injection of 3-methylcholanthrene (3MC). **b** Immunohistochemical detection of CD45 (green)-positive cells in skin sections 1 week after s.c. injection of 3MC. Black dots indicate melanin pigments in NMR dermis. Scale bar: 50 μm. Red arrowheads show positive cells in NMRs. Quantification of CD45-positive cells per area and/or total cells in skin sections at 1 (**c**), 3 (**d**), and 97 (**e**) weeks after s.c. injection of 3MC. **f** Schematic diagram for investigating immune cell infiltration into the skin after exposure to 7,12-dimethylbenz[a]anthracene (DMBA)/12-*O*-tetradecanoylphorbol-13-acetate (TPA). **g** Immunohistochemical detection of CD45 (green)-positive cells in skin sections at 2 weeks after exposure to DMBA/TPA. Scale bar: 50 μm. Red arrowhead shows a positive cell in NMRs. **h** and **i** Quantification of CD45-positive cells per area in skin sections at 2 (**h**), and 55 weeks (**i**) after exposure to DMBA/TPA. Mice were analysed at the end point (**i**). “Papilloma adjacent skin” is the no-papilloma region from DMBA/TPA-treated mouse skin. For quantification, data are presented as the mean ± SD of *n* = 3 (for **c**, mouse of **i**, NMR of **d** and **h**, control NMR of **e** and **i**), *n* = 4 (for control mouse of **d**, mouse of **h**) or *n* = 5 (for 3MC mouse of **d**, 3MC NMR of **e**, DMBA/TPA NMR of **i**) animals. Unpaired *t*-test versus untreated control (Ctrl).

The effect of chemical carcinogens on immune cell infiltration was evaluated by immunostaining using pre-validated antibodies against CD45 (leucocytes), IBA1 (macrophages), myeloperoxidase (MPO, myeloid cells: neutrophils and macrophages), and CD3 (T cells) (Supplementary Fig. 3 and Supplementary Table 1). In mice, 3MC treatment significantly increased the number of CD45-, IBA1-, and CD3-positive immune cells at 1 and 3 weeks relative to the total cell number or tissue area (Fig. 3a–d, Supplementary Fig. 4, and Supplementary Fig. 5a, b). These data reflect the infiltration of inflammatory immune cells after carcinogen treatment in mouse tissues as previously reported^16,17^. By contrast, 3MC-treated NMR skin showed low levels of immune cell infiltration. Although the number of inflammatory immune cells increased significantly after 3MC treatment, the total number was modulated to remain very low (Fig. 3a–d, Supplementary Fig. 4, and Supplementary Fig. 5a, b). Analysis of NMR skin at 97 weeks after 3MC treatment showed no significant increase in CD45-positive immune cells (Fig. 3a, e and Supplementary Fig. 5c).

Similar to the results regarding the effects of 3MC, DMBA/TPA-treated NMR skin at 2 weeks showed a very small increase in the number of several immune cell types in contrast with mice (Fig. 3f–h and Supplementary Fig. 6a, b). The accumulation of immune cells was markedly attenuated in NMR skin after 55 weeks of DMBA/TPA treatment, including after 108 rounds of treatment with TPA, a potent inflammatory agent (Fig. 3i and Supplementary Fig. 6c). These results indicate that the infiltration of inflammatory immune cells was much lower in NMRs than in mice after exposure to two types of chemical carcinogens.

Furthermore, we evaluated skin tissue responses to UV irradiation, which promotes carcinogenesis by inducing DNA damage and inflammation in mice^16,25^ (Supplementary Fig. 7a). UVB irradiation significantly increased skin thickness, the number of pH2AX-positive DNA-damaged cells, TUNEL-positive dead cells, and cleaved caspase-3-positive apoptotic cells in both mouse and NMR skin, indicating that tissue damage increases in both species (Supplementary Fig. 7b, c). In mouse skin, UV irradiation resulted in significant increases in the numbers of CD45-, IBA1-, and MPO-positive immune cells, whereas in NMR skin, UV irradiation resulted in very small increases in the number of CD45- and CD3-positive immune cells, and no significant increase in IBA1- or MPO-positive immune cells (Supplementary Fig. 8). These results indicate that inflammatory immune cell accumulation in response to various cancer-promoting stimuli is attenuated in NMRs, despite the induction of DNA damage and cellular responses such as cell death and proliferation.

Next, we evaluated infection-associated inflammatory responses in NMRs. Subcutaneous injection of bacterial lipopolysaccharide (LPS), which reportedly activates NMR immune cells^26^, increased interleukin-6 (*IL6*) expression, as well as the number of CD45- and MPO-positive immune cells in both mouse and NMR skin (Supplementary Fig. 9). Intraperitoneal LPS injection significantly increased the number of IBA1-positive immune cells in NMR livers (Supplementary Fig. 10a). Thus, NMR immune cells can infiltrate into tissues in response to bacterial virulence factors. When co-cultured with dead NMR fibroblasts, NMR macrophages exhibited normal phagocytic activity of dead cells (Supplementary Fig. 10b) despite showing reduced infiltration into carcinogen-treated tissues.

During these experiments, we observed that the number of immune cells was lower in control NMR skin than in control mouse skin. To provide a context for this, we examined the number of tissue-resident immune cells in various tissues from mice, rats, guinea pigs, and NMRs. We found that the number of IBA1- or CD3-positive immune cells was lower in NMR skin and intestine tissues, suggesting unique tissue immune homoeostasis in NMRs (Supplementary Fig. 11).

To investigate the overall inflammatory responses to 3MC, UV, and LPS treatment, we performed global gene expression analysis using RNA-sequencing (RNA-seq). Changes in global gene expression or in selected ligand genes important for cell-to-cell communication, including many chemokines and cytokines^27^, in response to the different treatments were greater in mouse skin than in NMR skin, and the upregulations were particularly large in the 3MC- and UV-treated mouse groups (Supplementary Fig. 12a, b and Supplementary Data 1). Cell type enrichment analysis using xCell^28^ showed that all treatments significantly increased several immune cell enrichment scores in mouse skin (Supplementary Fig. 12c and Supplementary Data 2). By contrast, in NMR skin, 3MC and UV treatment did not significantly change the immune cell enrichment scores, whereas LPS did (Supplementary Fig. 12c). These results are consistent with those of immunohistochemical analyses and western blotting of immune cell markers (Fig. 3 and Supplementary Figs. 4–6, 8, 9, 12d, e), and confirm that inflammatory responses to cancer-promoting stimuli are attenuated in NMR tissues.

### Loss-of-function mutations in necroptosis regulators in NMRs may contribute to the attenuated inflammatory response and carcinogenesis resistance

**Fig. 4:**
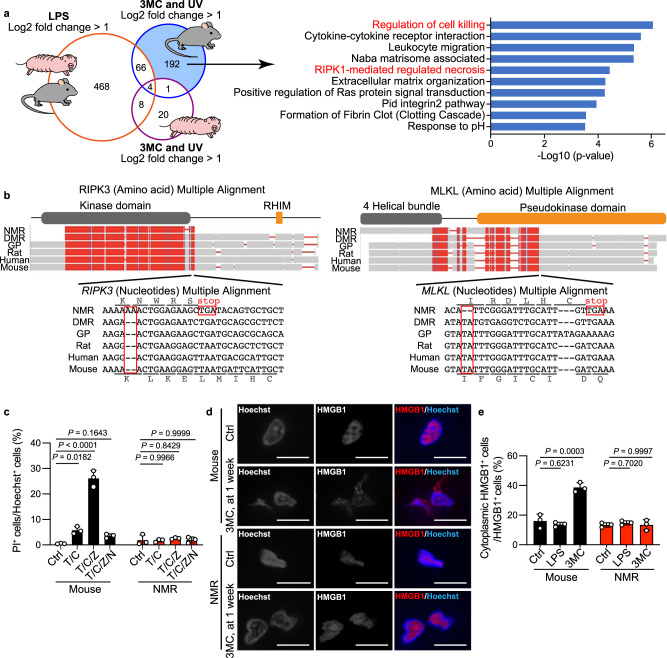
Loss of necroptosis regulators in naked mole-rats (NMRs). **a** Venn diagram showing the number of genes upregulated in both mouse and NMR skin upon lipopolysaccharide (LPS) treatment; genes upregulated specifically in mouse or NMR skin upon exposure to 3-methylcholanthrene (3MC, 1 week) and UV; and enriched pathways of 3MC-UV Mouse-DEGs. **b** Multiple alignments of receptor-interacting kinase 3 (*RIPK3*) and mixed lineage kinase domain-like (*MLKL*) sequences from the NMR, Damaraland mole-rat (DMR), guinea pig (GP), rat, human, and mouse. Frame-shift mutations and premature stop codons in the NMR sequence are boxed. Reading frames for the NMR and mouse sequences are indicated. The functional domains are shown above the alignments. **c** Cell death analysis in fibroblasts treated with a combination of TNF-α (T), cycloheximide (C), z-VAD-fmk (Z), or Nec-1 (N). Data are presented as the mean ± SD of *n* = 3 independent experiments. **d** Immunofluorescence staining of high mobility group box-1 protein (HMGB1) in skin at 1 week after 3MC-injection. Scale bar: 10 μm. **e** Quantification of cytoplasmic HMGB1 in skin after each treatment. Data are presented as the mean ± SD of *n* = 3 animals for each species. One-way ANOVA with Tukey’s multiple comparison test for (**c**) and Dunnett’s multiple comparisons test versus untreated control (Ctrl) for (**e**).

To examine the mechanisms underlying the different responses of NMR tissues to cancer-promoting stimuli, we analysed differentially expressed genes (DEGs) in response to 3MC and UV treatment that differed from DEGs in response to LPS treatment between mice and NMRs. We selected genes that were species-specifically upregulated by >2 fold in NMRs or mice after both 3MC and UV treatment, but that were not commonly upregulated after LPS treatment (Fig. 4a, blue-filled area, collectively termed 3MC-UV Mouse-DEGs and Supplementary Fig. 13a, purple-filled area, collectively termed 3MC-UV NMR-DEGs). Enrichment analysis of the selected DEGs was performed using Metascape^29^. Among 3MC-UV NMR-DEGs, genes related to the Kyoto Encyclopedia of Genes and Genomes (KEGG) pathway “p53 signalling pathway” were highly enriched, suggesting activation of the p53 pathway in 3MC- and UV-treated NMR skin (Supplementary Fig. 13a and Supplementary Table 2). This was consistent with the immunostaining data showing increased DNA damage and cell death in 3MC- and UV-treated NMR skin (Fig. 1e, Supplementary Fig. 1f, and Supplementary Fig. 7b, c). Among 3MC-UV Mouse-DEGs, genes related to the KEGG pathway “Cytokine-cytokine receptor interaction” and the gene ontology (GO) term “Leucocyte migration” were highly enriched, indicating the activation of inflammatory responses in 3MC- and UV-treated mouse skin (Fig. 4a and Supplementary Table 2).

Notably, among 3MC-UV Mouse-DEGs, genes related to the KEGG pathways “RIPK1-mediated regulated necrosis” (necroptosis) and “Regulation of cell killing” were the most significantly enriched (Fig. 4a and Supplementary Table 2), which were not observed among 3MC-UV NMR-DEGs. Necroptosis, a type of programmed necrotic cell death, triggers inflammation, and promotes colon, pancreatic, and liver cancer development^14,30–33^. Thus, we hypothesised that inactivation of necroptosis in NMRs may underlie the attenuated inflammatory responses in NMRs, possibly leading to cancer resistance.

The RIPK1-RIPK3 complex induces necroptosis via the necroptosis effector, MLKL^34–36^. We found that the NMR genome harbours a two-nucleotide insertion in the *RIPK3* gene and a two-nucleotide deletion in the *MLKL* gene, which causes frame-shift mutations and introduces premature stop codons (Fig. 4b). These alterations remove the RHIM domain in RIPK3 and the pseudokinase domain in MLKL, which are both functionally essential for inducing necroptosis in other mammalian species^37^. Because NMR *RIPK3* and *MLKL* genes have premature stop codons located before the final exon, the transcripts from these two genes are putative targets for nonsense-mediated mRNA decay (NMD)^38^. As NMR *RIPK3* mRNA was expressed in the skin (Supplementary Fig. 13b, c), we examined whether NMR *RIPK3* is degraded by NMD. RT-qPCR analysis of NMR fibroblasts treated with actinomycin D (ActD, a transcriptional inhibitor) and/or cycloheximide (CHX, a translational inhibitor that potently inhibits NMD) showed that NMR *RIPK3* transcripts exhibited relatively low steady-state levels after ActD treatment, whereas *RIPK3* mRNA level increased upon CHX treatment (Supplementary Fig. 13d). This result indicates that NMR *RIPK3* mRNA is degraded by NMD. NMR *MLKL* mRNA expression was not detected in the skin (Supplementary Fig. 13e–g). Although previous studies have shown that only the N-terminal 4-alpha helical bundle domain of MLKL can cause spontaneous cell death depending on the cellular context^39–41^, NMR MLKL could not induce spontaneous cell death (Supplementary Fig. 14a, b). Thus, the genes essential for necroptosis induction are likely to be defective in NMRs.

To evaluate whether necroptosis is impaired in NMRs, we performed experimental necroptosis induction in vitro. In mouse fibroblasts, treatment with tumour necrosis factor-α (TNF-α), CHX, and z-VAD-fmk (caspase inhibitor) caused massive cell death, which was inhibited by necrostatin-1 (Nec1, RIPK1 inhibitor), as previously reported^42^, indicating activation of necroptosis. In contrast to mouse fibroblasts, NMR fibroblasts did not show increased cell death in response to TNF-α + CHX or TNFα + CHX + z-VAD-fmk, although TNF-α upregulated *IL6*^43^ as observed in mice (Fig. 4c and Supplementary Fig. 14c). These results suggest that NMR cells are incapable of inducing TNF-α-mediated necroptosis and apoptosis, although they are capable of inducing DNA damage-induced caspase-3-dependent apoptosis (Supplementary Fig. 14d–f). In mice, RIPK3 is important for the induction of both necroptosis and TNF-induced apoptosis mediated by RIPK1^44^. Thus, loss-of-function mutation of *RIPK3* in NMRs may contribute to their inability to undergo necroptosis and TNF-induced apoptosis mediated by RIPK1.

Generally, necroptosis triggers inflammation through the release of various cellular components such as high mobility group box-1 protein (HMGB1)^45^, which can be observed during cancer progression^16,17^. 3MC, DMBA, and UV treatment did not significantly alter cytoplasmic HMGB1 translocation in NMR skin, in contrast to the significant increase observed in mouse skin (Fig. 4d, e and Supplementary Fig. 15). These results further support the idea that the inability to induce necroptosis in NMRs may contribute to the dampened immune cell responses to carcinogenic stimuli.

**Fig. 5:**
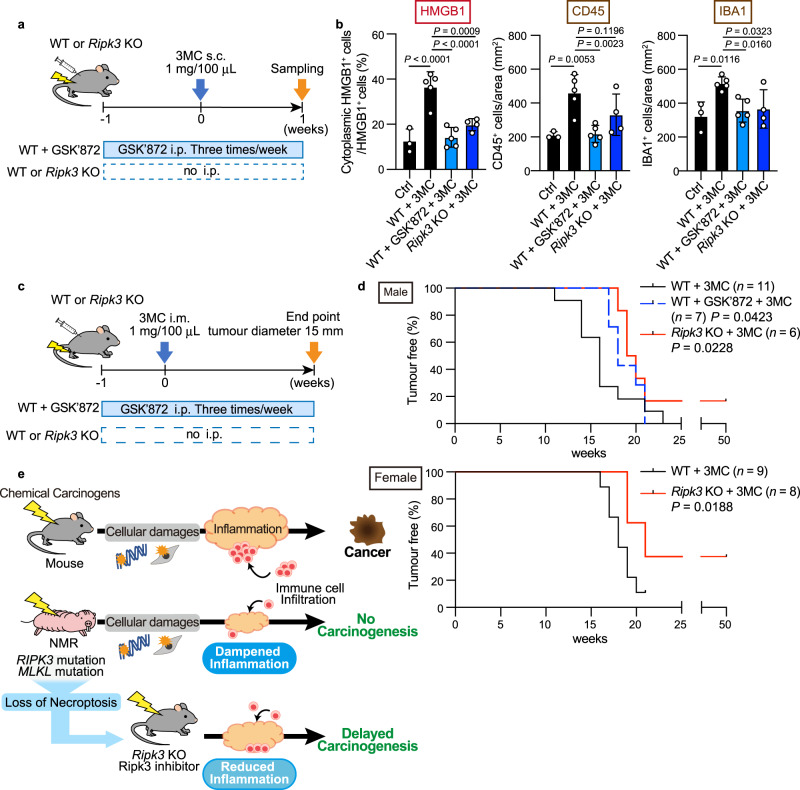
Inhibition or disruption of Ripk3 attenuates tissue inflammatory response and delays chemical carcinogenesis in mice. **a** Schematic diagram for investigating responses to subcutaneous (s.c.) 3-methylcholanthrene (3MC) injection with intraperitoneal (i.p.) injection of GSK’872 in *Ripk3* wild-type (WT) mice or s.c. 3MC injection in *Ripk3* knockout (KO) mice. **b** Quantification of cytoplasmic HMGB1-, CD45-, and Iba1-positive cells in mouse skin after exposure to 3MC with or without GSK’872 in WT or *Ripk3* KO mice. Data are presented as the mean ± SD of *n* = 3 (for control (Ctrl)), *n* = 4 (for *Ripk3* KO + 3MC), or *n* = 5 (for WT + 3MC and WT + GSK’872 + 3MC) animals. One-way ANOVA with Dunnett’s multiple comparisons test versus WT + 3MC. **c** Schematic diagram for carcinogenesis induction by intramuscular (i.m.) injection of 3MC with i.p. injection of GSK’872 in mice or i.m. injection of 3MC in *Ripk3* KO mice. **d** Kaplan–Meier curves of tumour-free mice (*n* = 11 [for wild-type, WT], *n* = 7 [for GSK’872], or *n* = 6 [for *Ripk3* KO] animals for male and *n* = 9 [for WT] or *n* = 8 [for *Ripk3* KO] animals for female). *P* = 0.0423 for GSK’872 and *P* = 0.0228 for *Ripk3* KO for male and *P* = 0.0188 for *Ripk3* KO for female; Gehan–Breslow–Wilcoxon test. **e** Graphical illustration which shows the main finding from this paper. After carcinogenic treatments, DNA damage and cellular responses, such as cell death and proliferative changes, are induced in both NMRs and mice; however, only NMRs show attenuated inflammatory responses and do not develop tumours. Inhibition or disruption of Ripk3 in mice resulted in the reduced inflammatory response and the delayed onset of chemical carcinogenesis. Thus, in NMRs, loss-of-function mutations in genes essential for necroptosis induction may attenuate inflammatory responses and serve as an in vivo cancer resistance mechanism.

Next, we assessed whether inhibition of necroptosis by RIPK3 inhibition could suppress the 3MC-induced inflammatory response and impede carcinogenesis in mice. Specifically, we used a RIPK3 inhibitor GSK’872^46^ or *Ripk3* gene disruption (Fig. 5a and Supplementary Figs. 16, 17a). The results of western blotting showed that 3MC treatment activated the MLKL protein (as indicated by MLKL phosphorylation), and MLKL activation was suppressed by GSK’872 treatment (Supplementary Fig. 17b). GSK’872 and disruption of the *Ripk3* gene significantly suppressed cytoplasmic HMGB1 translocation after exposure to 3MC (Fig. 5b and Supplementary Fig. 17c), indicating that necroptosis was successfully suppressed. In addition, both manipulations reduced the infiltration of inflammatory immune cells in 3MC-treated mouse skin (Fig. 5b and Supplementary Fig. 17d). Finally, we evaluated the effect of GSK’872 treatment or *Ripk3* knockout on 3MC-induced chemical carcinogenesis in mice (Fig. 5c). Continuous administration of GSK’872 or disruption of the *Ripk3* gene significantly delayed the onset of carcinogenesis in 3MC-treated mice (Fig. 5d; *P* = 0.0423 for GSK’872; *P* = 0.0228 for *Ripk3* KO male mice; *P* = 0.0188 for *Ripk3* KO female mice; Gehan–Breslow–Wilcoxon test). Four out of 14 *Ripk3* KO mice did not develop tumours for more than 50 weeks after 3MC treatment (Fig. 5d). Thus, in mice, the suppression of the necroptosis regulator attenuated immune cell infiltration and chemical carcinogenesis. This result is consistent with our assumption that an absence of necroptosis regulators in NMRs may contribute to the reduced inflammatory response and resistance to chemical carcinogenesis.

## Discussion

In this study, NMRs showed marked resistance to two types of chemical carcinogenesis induction in vivo. We revealed that a distinctive feature of the NMR tissue response to carcinogenic insults is an unusual dampened inflammatory response; The dampened inflammatory response may serve as a non-cell-autonomous cancer resistance mechanism in NMR individuals. In addition, Ripk3 disruption in mice resulted in reduced inflammatory response and delayed carcinogenesis. Therefore, we propose that NMR-specific loss-of-function mutations in the necroptosis regulators *RIPK3* and *MLKL* may be one of the mechanisms underlying the attenuated tissue inflammatory response and remarkable cancer resistance of NMRs (Fig. 5e).

In a different cancer-resistant rodent, the blind mole-rat, the same dose of 3MC causes low frequency carcinogenesis (~9%)^22,47^. Thus, NMRs are especially resistant to chemical carcinogenesis. In contrast to NMRs, 3MC induces massive inflammation in blind mole-rats^22^. This distinct difference in inflammatory responses between NMRs and blind mole-rats, both of which show spontaneous cancer resistance and a high DNA repair capacity^48–50^, may contribute to the differences in resistance to in vivo carcinogenesis induction.

The attenuated cancer-promoting tissue inflammatory response may act as a gatekeeper to prevent carcinogenesis in NMRs. This is supported by previous reports showing that toll-like receptor 4 knockout mice exhibit carcinogenic resistance owing to dampened inflammatory responses^16,17^. Other mechanisms besides the deficiency in necroptosis, especially those related to immune cell characteristics, might also contribute to the unique inflammatory response in NMRs. Immune homoeostasis in NMRs may be unusual because the resident immune cells, which contribute to the attenuated immune response and to cancer resistance in mice^51,52^, were less numerous in the skin and intestine tissues of NMRs than in those of other rodent species (Supplementary Fig. 11). Moreover, a single cell RNA-seq study of immune cells revealed the unique immune system of NMRs, which is characterised by a lack of natural killer cells^26^. Recent in silico and in vitro studies show that cancer-resistant bats lack certain immunity-related genes^53,54^. Future studies examining the immune system of cancer-resistant animals should improve our understanding of their cancer resistance mechanisms.

The type of cell death and its modulation play a critical role in the regulation of inflammation and homoeostasis in vivo. In this study, caspase-3-dependent apoptosis occurred in NMRs, whereas necroptosis did not (Fig. 4c–e, Supplementary Fig. 7b, c, and Supplementary Fig. 14d–f). Since the pro-inflammatory potential of necroptosis is markedly higher than that of apoptosis^45^, the suppression of necroptosis may contribute substantially to the attenuation of the inflammatory response in NMR tissues as in Ripk3 inhibited/disrupted mice. Necroptosis is involved in various inflammatory age-related diseases/disorders, such as ischaemia-reperfusion injury, atherosclerosis, and neurodegenerative diseases. Necroptosis also plays an important role in innate immunity during infectious diseases^19^. Notably, NMRs are resistant not only to cancer, but also to aging-related physiological declines, neurodegenerative disease, and ischaemia-reperfusion injury, while being highly susceptible to herpes virus infection^3,55–57^. It is possible that the deficiency in necroptosis induction may constitute an important part of the mechanisms responsible for the unusual disease susceptibility of the NMRs. It would also be interesting to study how other types of cell death, such as ferroptosis and pyroptosis, are regulated in NMRs. ﻿

Recent studies have shown that RIPK3 is involved not only in the induction of necroptosis and RIPK1-mediated apoptosis, but also in the activation of the NLRP3 inflammasome, maturation of IL-1β, and production of inflammatory cytokines, all of which are not directly activated via necroptosis^58,59^. MLKL contributes to various biological functions, such as endosomal trafficking and extracellular vesicle formation, in addition to the induction of necroptosis and inflammatory cytokines^60^. Therefore, the loss-of-function mutations of *RIPK3* and *MLKL* in NMRs may affect not only necroptosis, but also the attenuation of the tissue inflammatory response via suppression of the NLRP3 inflammasome and various other biological processes in vivo. This will require further analysis.

In addition to the role of cancer-promoting inflammation, the generation of mutant cells is also crucial for the initiation of carcinogenesis^61^. Although carcinogen treatment damaged DNA and cells in NMR skin (Figs. 1e, 2e, Supplementary Fig. 1f, and Supplementary Fig. 2a), it is possible that NMRs are protected against mutant cell generation or efficiently eliminate mutant cells. Possible explanations include (1) inhibition of mutant cell generation via several mechanisms, such as the previously reported efficient DNA double-strand break repair^49^, or (2) elimination of mutant cells by unknown mechanisms, which may synergistically contribute to in vivo cancer resistance of NMRs.

The present results shed light on the importance of studying not only NMR culture cells, but also tissues and individuals to gain mechanistic insights into the immune response and extraordinary carcinogenesis resistance of NMRs. Further insight into the tissue responses of the NMR to carcinogenic insults may lead to the development of new anticancer strategies for humans.

## Methods

### Animals

NMRs were maintained at Kumamoto University and Hokkaido University. All NMRs (8–31 months) used in this research were raised in rooms that were maintained at 30 ± 0.5 °C and 55 ± 5% humidity with 12 h light and 12 h dark cycles^10^. The NMRs used in this study are listed in Supplementary Table 3. Male C57BL/6N mice (8–10 weeks) were purchased from CLEA Japan, and *Ripk3* knockout (KO) mice were generated by deletion of the *Ripk3* gene. Wild-type mice and KO mice were kept in rooms that were maintained at 24.5 ± 1.5 °C and 50 ± 10% humidity with 12 h light and 12 h dark cycles. Male rats (Wistar, 6 months) and guinea pigs (Hartley, 6 months) were purchased from Japan SLC. The Ethics Committees of Kumamoto University (Approval no. A30-043 and A2020-042) and Hokkaido University (14-0065) approved all procedures, which were in accordance with the Guide for the Care and Use of Laboratory Animals (United States National Institutes of Health, Bethesda, MD, USA).

### Generation of *Ripk3* knockout mice and genotyping

*Ripk3* KO mice were generated by the introduction of the Cas9 protein (317–08441; NIPPON GENE), tracrRNA (GE-002; FASMAC), synthetic crRNA (FASMAC), and ssODN into C57BL/6N fertilised eggs by electroporation. For generating the *Ripk3* KO allele, the synthetic crRNAs were designed according to the sequence AAGAGAGACTGGCTATCGTG (GGG) of the 5′ upstream region of *Ripk3* and ACTAGGAGAGGATCCCACTG (AGG) in the *Ripk3* intron 9. The ssODN 5′- CGACTTTCTTTCGTTGTGTGACCTCAGttttatttGATAGCCAGTCTCTCTTGGACCCCTTAGCTCCACC-3′ was used as a homologous recombination template.

The electroporation solution contained 10 μM tracrRNA, 10 μM synthetic crRNA, 0.1 μg per μL Cas9 protein, and 1 μg per μL ssODN in Opti-MEM I Reduced Serum Medium (31985062; Thermo Fisher Scientific). Electroporation was performed using the Super Electroporator NEPA 21 (NEPA GENE) on glass microslides with round wire electrodes (1.0 mm gap [45–0104; BTX]). Four steps of square pulses were applied (1], three times of 3 mS poring pulses with 97 mS intervals at 30 V; 2], three times of 3 mS polarity-changed poring pulses with 97 mS intervals at 30 V; 3], five times of 50 mS transfer pulses with 50 mS intervals at 4 V with 40% decay of voltage per pulse; 4], five times of 50 mS polarity-changed transfer pulses with 50 mS intervals at 4 V with 40% decay of voltage per pulse).

The targeted *Ripk3* KO allele in F0 mice was identified by genomic PCR using the following primers: *Ripk3* KO F: 5′- AGCGACACCTTGTGATCTCC-3′ and *Ripk3* KO R: 5′- CTGGCCCAAGACAACCCTTA -3′ for the knockout allele (396 bp); *Ripk3* Wild F: 5′- GGAAAAGTCAGCCAATCCCG -3′ and *Ripk3* Wild R: 5′- GCAAGACTAGAGCACACCCTC -3′ for the wild-type allele (375 bp).

### 3MC treatment

C57BL/6N mice (average body weight, 24.0 g), NMRs (average body weight, 26.6 g), and *Ripk3* KO mice were intramuscularly or subcutaneously injected with 3MC (Sigma-Aldrich; 1 mg dissolved in 100 μL corn oil) into the hindlimbs or back skin^62^. Animals were observed weekly until tumours >15 mm in diameter developed at the injected sites, at which point the animals were sacrificed humanely using isoflurane anaesthesia, and the tumours were used for further analysis. For NMRs, muscle samples (3MC-injected sites and opposite sites as controls) collected after 114 weeks and skin samples collected after 97 weeks were used for histopathological analysis. One group of NMRs received the same concentration of 3MC per body weight as mice (1–1.52 mg, 41.7 µg per g body weight of 3MC). After 49 weeks, no tumours developed in NMRs.

To evaluate the responses to short exposure to 3MC, 3MC (1 mg dissolved in 100 μL corn oil) was subcutaneously injected into the back skin of C57BL/6N mice or NMRs, and the site was examined at 1 or 3 weeks after injection. Injected sites (100 mm^2^) were collected and used for further analysis. To suppress RIPK3 activity, GSK’872^27,41^ (SelleckBio; 1 mg per kg body weight dissolved in saline) was intraperitoneally injected three times a week from 1 week before 3MC treatment until the end point of the experiment.

### DMBA/TPA treatment

C57BL/6N mice and NMRs were treated with DMBA (Sigma-Aldrich; 100 μg in 100 μL acetone) on the back skin. One week after DMBA treatment, animals were treated twice a week with TPA (Cayman Chemical; 12.5 μg in 100 μL acetone) until tumour formation was observed^63^. Animals were observed daily until tumours >7 mm in diameter developed on the skin, at which point the animals were sacrificed humanely by isoflurane anaesthesia, and the tumours were used for further analysis. For NMRs, skin biopsies were performed under isoflurane anaesthesia at 55 weeks, and samples were used for histopathological analysis. For histopathological analysis, one individual that had an external wound possibly due to fighting was excluded as previously described^24^.

To evaluate responses to short exposure to DMBA, DMBA (100 μg in 100 μL acetone) was administered to the back skin, and skin biopsies were performed after 24 h. To evaluate responses to short exposure to DMBA/TPA, DMBA (100 μg in 100 μL acetone) was administered to the back skin, and 1 week after DMBA treatment, animals were subsequently treated three times with TPA (12.5 μg in 100 μL acetone). Skin biopsies were performed 1 week after starting TPA treatment.

### Haematoxylin and eosin (HE)-staining and immunohistochemical analysis

Histological examination was performed at K.I. Stainer, Inc. (Kumamoto, Japan). Briefly, the samples were fixed in 4% paraformaldehyde in phosphate-buffered saline (PBS), embedded in paraffin, and cut into 4 μm sections; HE staining was routinely performed. The antibodies and protocols are listed in Supplementary Table 1. Briefly, for immunostaining, the sections were deparaffinised using xylene and rehydrated with a graded series of ethanol. Antigen retrieval was performed by heat-induced epitope retrieval in citrate buffer or Tris buffer, or by enzymatic retrieval using proteinase K^64^. The sections were incubated with 1% bovine serum albumin in Tris-buffered saline with 0.1% NaN_3_ for blocking, and stained with primary antibodies against CD45 (Abcam, ab10558), MPO (DAKO, A0398), IBA1 (FUJIFILM WAKO, 019-19741), CD3 (Nichirei, 413591), Ki67 (Abcam, ab16667), 8-OHdG (Santa Cruz Biotechnology, sc-393871), pH2AX (Cell Signalling Technology [CST], 9718), or HMGB1 (Abcam, ab79823). The sections were incubated with horseradish peroxidase (HRP)‐conjugated anti-rabbit, anti-mouse, or anti-rat secondary antibodies (Nichirei) as a secondary antibody. Positive signals were visualised using HistoGreen substrate (Cosmo Bio) for staining of immune cells in the skin (because it is not easy to distinguish diaminobenzidine (DAB)-stained cells from dermal melanin pigments in NMR skin in limited sized figures) or DAB (Nichirei). For HMGB1, Alexa Fluor 555 anti-rabbit IgG (CST, A21429) secondary antibody was used. Nuclei were counterstained with haematoxylin (for CD45, MPO, IBA1, CD3, Ki67, 8-OHdG, and pH2AX) or Hoechst 33258 (Sigma-Aldrich) for HMGB1.

For cleaved caspase-3, 10 μm fresh-frozen sections were fixed with 4% PFA, washed with PBS, and blocked with 5% normal goat serum in 0.3% Triton X-100 (Nacalai Tesque) in PBS. The sections were incubated with primary antibodies against cleaved caspase-3 (CST; 9664; 1:400) in Can Get Signal Solution B (TOYOBO). The sections were stained with Alexa Fluor 555 anti-rabbit IgG (CST; A21429; 1:1000) as a secondary antibody, and nuclei were stained with 1 μg per mL Hoechst 33258 (Sigma-Aldrich).

The images were captured using a BZ-X 710 fluorescence microscope (KEYENCE) and analysed using a BZ-X image analyser (KEYENCE).

### TUNEL staining

For TUNEL staining, 4 μm paraffin sections were deparaffinised and rehydrated as described above. The sections were stained using the TUNEL Assay Kit BrdU-Red (Abcam) according to the manufacturer’s instructions. Nuclei were counterstained with Hoechst 33258. The images were captured using a BZ-X 710 fluorescence microscope and analysed using a BZ-X image analyser (KEYENCE).

### Morphometric analyses of skin inflammatory responses

Epidermal thickness was quantified by calculating the mean length of skin surface to the epidermal junction by five hand-drawn line segments per field (four fields were analysed per animal) using ImageJ. Positive cells identified by immunostaining and TUNEL staining were quantified by counting the mean number of cells in each of the four images of one section from more than three animals per experiment, and were normalised to the total number of cells (for Ki67, TUNEL, pH2AX, 8-OHdG, cleaved caspase-3, CD45, IBA1, MPO, and CD3) or to the tissue area (for CD45, IBA1, MPO, and CD3). The quantification was performed by three independent investigators including a pathologist (Y. Komohara). Total cells, either Hoechst or haematoxylin-positive nuclei (at least 350 cells per animal) and tissue area (bright field), were measured using a Hybrid Cell Count application (KEYENCE) in a BZ-X image analyser.

Cytoplasmic HMGB1-positive cells were quantified by counting the mean number of cells in highly magnified sections from more than three animals per experiment, and normalised to the number of total HMGB1-positive cells (at least four images from one section per animal, >100 cells). The quantification was performed by two independent investigators.

### UV irradiation

UV irradiation on the back skin of C57BL/6N mice and NMRs was performed every other day for 12 days with a dose of 1000 J m^−2^ using a UV lamp (UVP UVM-28; Analytic Jena) for the times indicated in Supplementary Fig. 7a^25^. Prior to irradiation, the back skin of C57BL/6N mice was shaved. At 24 h after final irradiation, the animals were sacrificed humanely using isoflurane anaesthesia, and the skin samples were used for further analysis.

### LPS treatment

C57BL/6N mice and NMRs were subcutaneously or intraperitoneally injected with LPS (Sigma-Aldrich; 10 mg per kg body weight dissolved in saline). After 24 h of treatment, the animals were sacrificed humanely using isoflurane anaesthesia, and skin or liver samples were used for further analysis.

### RNA isolation and quantification of gene expression

Total RNA was extracted using the RNeasy Plus Mini Kit (Qiagen, for cells) or TRIzol (Thermo Fisher Scientific, for tissues) according to the manufacturer’s protocol. The gDNA Eliminator Spin Column (Qiagen) or the TURBO DNA-free™ Kit (Invitrogen) was used for genomic DNA elimination according to the manufacturer’s protocol. Reverse transcription reactions were performed with ReverTra Ace qPCR RT Master Mix (TOYOBO) using 300 ng total RNA as a template. The resulting cDNA was used for reverse transcription polymerase chain reaction (RT-PCR) and quantitative reverse transcription PCR (RT-qPCR). For RT-PCR, 24 cycles (for *actin beta* [*ACTB*]) or 35 cycles (for *MLKL*) of amplification were performed under the following conditions using PrimeSTAR Max DNA Polymerase (Takara): denaturing at 98 °C for 10 s, annealing at 55 °C for 30 s, and extension at 72 °C for 30 s. The DNA fragments were electrophoresed in 2% agarose gels. RT-qPCR analysis was performed using Thunderbird SYBR qPCR Mix (TOYOBO) or PowerUp SYBR Green Master Mix (Thermo Fisher Scientific) on a CFX384 Touch Real-Time PCR Detection System (Bio-Rad) with the primers listed in Supplementary Table 4^65^.

### Cell culture

Primary NMR or mouse skin fibroblasts were obtained from the back skin of 1–2-year-old NMRs or 6–8-week-old C57BL/6N mice^10^. The cells were cultured in Dulbecco’s modified Eagle’s medium (Sigma-Aldrich) supplemented with 15% foetal bovine serum (FBS) (for NMR fibroblasts) or 10% FBS (for mouse fibroblasts) (Gibco), 1% penicillin/streptomycin (FUJIFILM WAKO), 2 mM L-glutamine (FUJIFILM WAKO), and 0.1 mM non-essential amino acids (FUJIFILM WAKO) at 32 °C in a humidified atmosphere containing 5% O_2_ and 5% CO_2_. We used the fibroblasts within five passages. The medium was replaced every 2 days. For investigation of NMD, NMR fibroblasts were incubated with 5 μg per mL ActD (Sigma-Aldrich) and/or 30 μg per mL CHX (FUJIFILM WAKO) for 4 h. NMR fibroblasts treated with DMSO served as the control. After treatment, total RNA was isolated and used for RT-qPCR as described above.

### Lentiviral overexpression of NMR-MLKL

Because NMR-*MLKL* mRNA was not expressed in NMR skin, the coding sequence of NMR-*MLKL* was artificially synthesised based on the NCBI sequence information and our genomic sequencing results (XM_021256495.1 and Fig. 4b) (Eurofins Genomics) and inserted into the lentiviral vector pCSII-EF-RFA-hyg (kindly provided by H. Naka-Kaneda). Then, the pCSII-EF-NMR-MLKL plasmid and packaging vectors (pCMV-VSV-G-RSV-Rev and pCAG-HIVgp)^66^ were used to transfect 293T cells using a polyethylenimine MAX transfection reagent (CosmoBio) according to the manufacturer’s instructions. The conditioned medium containing viral particles was concentrated by ultracentrifugation and used for viral transduction into NMR SV40ER cells, a NMR skin fibroblast cell line expressing simian virus 40 early region^67^. The transduced cells were passaged and subjected to necroptosis assays and propidium iodide (PI) staining.

### Necroptosis assay

Primary mouse or NMR fibroblasts and NMR SV40ER cells were seeded at 1 × 10^4^ cells per well onto 24-well plates and stimulated with TNF-α (PeproTech; 50 ng per mL), z-VAD-fmk (Abcam; 20 μM), CHX (1 μg per mL) and Nec-1 (Sigma-Aldrich; 20 μM). After 24 h, cells were stained with Hoechst 33342 (DOJINDO; 1 μg per mL) for 10 min at 32 °C. Then, the cells were stained with PI (FUJIFILM Wako; 10 μg per mL) for 5 min at 32 °C. Images were captured using a BZ-X 710 fluorescence microscope (KEYENCE), and the number of cells positive for PI or Hoechst 33342 was counted (at least 100 cells per treatment) using a BZ-X image analyser (KEYENCE). PI and Hoechst 33342 double-positive cells were regarded as dead cells.

### Etoposide treatment

NMR fibroblasts were exposed to etoposide at 200 μM for 4 days. Etoposide-containing medium was added to subconfluent fibroblasts. After 2 days, the medium was replaced by freshly prepared etoposide-containing medium for an additional 2 days. Then, the cells were collected for Annexin V/PI analysis and western blotting.

### Flow cytometry analysis for apoptosis detection

The FITC Annexin V Apoptosis Detection Kit (BD Biosciences or BioLegend) was used for the detection of apoptosis. Primary NMR skin fibroblasts were stained according to the manufacturer’s protocols and analysed on a FACSVerse (BD Biosciences) flow cytometer.

### Phagocytosis assay

NMRs and mice were sacrificed humanely using isoflurane anaesthesia, and limbs were isolated. After removing muscles and cartilage tissue, the bones were crushed and suspended in PBS. The cell suspension was filtered through a 70 μm cell strainer (Falcon) and suspended in hypo-osmotic solution to remove red blood cells. The remaining cells after haemolysis were processed into a single cell suspension and cultured in RPMI-1640 (FUJIFILM WAKO) supplemented with 15% FBS, 1% penicillin/streptomycin, 2 mM L-glutamine, 0.1 mM non-essential amino acids, and 20 ng per mL mouse macrophage colony stimulating factor (M-CSF) (BioLegend) for 8 days^64^. Dead cells were prepared by 200 J m^−2^ of UVC irradiation to fibroblasts using a UV crosslinker (Analytic Jena). After UV irradiation, cells were cultured for 24 h, and dead cells were collected and stained using pHrodo (Thermo Fisher Scientific) according to the manufacturer’s protocol. The same amounts of pHrodo-labelled dead cells (5 × 10^5^ cells) were co-incubated with NMR or mouse bone marrow macrophage culture. After 2 h, phagocytosis was evaluated by measuring pH-sensitive fluorescence of pHrodo using the BZ-X image analyser (KEYENCE).

### RNA-seq analysis

Total RNA was extracted from mouse and NMR skin tissues using TRIzol (Thermo Fisher Scientific) and purified using the RNeasy Plus Mini Kit (Qiagen); likely contaminated genomic DNA was removed from total RNA using the RNase-Free DNase Set (Qiagen) according to the manufacturer’s protocol. cDNA libraries were generated from 200 ng total RNA using a TruSeq stranded mRNA library preparation kit (Illumina). The resultant libraries were sequenced on NextSeq550 (Illumina) in single-ended mode. Low-quality bases and the adapters in the sequenced reads were trimmed using Cutadapt (ver.1.14)^68^ with Python 2.7.6. The trimmed reads were mapped to either the mouse (mm10) or NMR (HetGla_female_1.0) reference genome, with the UCSC refGene gtf for mouse and the Ensembl HetGla gtf and previously published gff^69^ for NMR, using STAR (ver.2.4.1d)^70^. For identification of DEGs, the uniquely mapped reads were counted and normalised to calculate fold changes and false discovery rate (FDR) using HTSeq (ver.0.11.2)^71^ and edgeR (ver.3.18.1)^72^, with the UCSC refGene gtf for mouse and the Ensembl HetGla gtf and previously published gff^69^ for NMR. Enrichment of genes in specific cellular functions (GO terms, Reactome, and KEGG pathways) was analysed using Metascape^29^. The gene expression levels were calculated as transcripts per million (TPM) using deepTools (ver.2.1.0)^73^, and mapping was visualised using the Integrative Genomics Viewer. The immune enrichment score was analysed using xCell^28^.

### Western blotting

The skin or cell samples were lysed in cell-lysis buffer (125 mM Tris-HCl, pH 6.8, 4% sodium dodecyl sulphate [SDS], and 10% sucrose) and boiled for 10 min. Protein concentration was measured using the BCA Protein Assay Kit (Takara Bio). The protein samples were subjected to SDS-polyacrylamide gel electrophoresis and transferred to polyvinylidene fluoride membranes using the Trans-Blot Turbo Transfer System (Bio-Rad). Membranes were probed with antibodies against MLKL (Abcam, ab184718; 1:1000), pMLKL (Abcam, ab196436; 1:1000), CD45 (Abcam, ab10558; 1:500), cleaved caspase-3 (CST; 9664; 1:1000), β-actin (CST; 4970; 1:2000), GAPDH (Invitrogen, MA5-15738; 1:1000), or vinculin (Sigma-Aldrich, V9131; 1:1000). The membranes were incubated with HRP-conjugated anti-rabbit (CST, 7074; 1:1000) or HRP-conjugated anti-mouse (CST, 7076; 1:1000) IgG secondary antibodies and visualised using ECL Prime Western Blotting Detection Reagent (GE Healthcare) and ImageQuant LAS 4000 Mini (FUJIFILM). The relative band intensity of CD45 relative to GAPDH was calculated using MultiGauge (FUJIFILM). The experiments were performed in biological duplicates or triplicates.

### Statistics and reproducibility

We used GraphPad Prism (GraphPad ver.8) for statistical analysis. The two groups were analysed using the two-tailed unpaired *t*-test. For multiple comparisons, the data were analysed using one-way analysis of variance (ANOVA), followed by Tukey’s multiple comparisons test for multiple comparisons or by Dunnett’s multiple comparisons test. Time to tumour progression was estimated using Kaplan–Meier curves and was statistically analysed using the log-rank Mantel–Cox test or the Gehan–Breslow–Wilcoxon test. Each data point represents the mean ± standard deviation (SD) derived from at least three animals or biological replicates. *P*-values < 0.05 were considered statistically significant. The sample sizes and the number of replicates (at least three individuals or three independent experiments) were described in the figure legends.

### Reporting summary

Further information on research design is available in the Nature Research Reporting Summary linked to this article.

## Supplementary information

Supplementary Information

Description of Additional Supplementary Files

Supplementary Data 1

Supplementary Data 2

Supplementary Data 3

Reporting summary

## Data Availability

RNA-seq data are deposited in the DDBJ under the accession number DRA010882. Uncropped versions of western blots and gels are provided as Supplementary Figs. 18 and 19. The source data underlying the graphs and charts in the main manuscript are provided as Supplementary Data 3. Other data supporting the findings of this study are available from the corresponding author upon reasonable request.
