# Supplementary material for: Resistance to chemical carcinogenesis induction via a dampened inflammatory response in naked mole-rats
Source: Commun Biol. 2022 Mar 30;5:287. doi: 10.1038/s42003-022-03241-y (PMC8967925; doi:10.1038/s42003-022-03241-y)
Supplement: Supplementary file 1 — Supplementary Information [file 42003_2022_3241_MOESM1_ESM.pdf]

Resistance to chemical carcinogenesis induction via a dampened inflammatory response in naked mole-rats

Kaori Oka[1,2†], Shusuke Fujioka[1,2†], Yoshimi Kawamura[1,2†], Yoshihiro Komohara[3], Takeshi Chujo[4], Koki Sekiguchi[1], Yuki Yamamura[1], Yuki Oiwa[1,2], Natsuko Omamiuda-Ishikawa[1], Shohei Komaki[5], Yoichi Sutoh[5], Satoko Sakurai[6], Kazuhito Tomizawa[4,7], Hidemasa Bono[8], Atsushi Shimizu[5,9], Kimi Araki[7,10], Takuya Yamamoto[6,11,12,13], Yasuhiro Yamada[13,14], Hiroyuki Oshiumi[15], and Kyoko Miura[1,2,7*]

1) Department of Aging and Longevity Research, Faculty of Life Sciences, Kumamoto University, Kumamoto 860-0811, Japan.

2) Biomedical Animal Research Laboratory, Institute for Genetic Medicine, Hokkaido University, Sapporo 060-0815, Japan.

3) Department of Cell Pathology, Faculty of Life Sciences, Kumamoto University, Kumamoto 860-8556, Japan.

4) Department of Molecular Physiology, Faculty of Life Sciences, Kumamoto University, Kumamoto 860-8556, Japan.

5) Division of Biomedical Information Analysis, Iwate Tohoku Medical Megabank Organization, Disaster Reconstruction Center, Iwate Medical University, Iwate 028-3694, Japan.

6) Department of Life Science Frontiers, Center for iPS Cell Research and Application (CiRA), Kyoto University, Kyoto 606-8507, Japan.

7) Center for Metabolic Regulation of Healthy Aging, Kumamoto University, Kumamoto 860-8556, Japan.

8) Program of Biomedical Science, Graduate School of Integrated Sciences for Life, Hiroshima University, Hiroshima 739-0046, Japan.

9) Division of Biomedical Information Analysis, Institute for Biomedical Sciences, Iwate Medical University, Iwate 028-3694, Japan.

10) Institute of Resource Development and Analysis, Kumamoto University, Kumamoto 860-0811, Japan.

11) Institute for the Advanced Study of Human Biology (WPI-ASHBi), Kyoto University, Kyoto 606-8501, Japan.

12) Medical-risk Avoidance based on iPS Cells Team, RIKEN Center for Advanced Intelligence Project (AIP), Kyoto 606-8507, Japan.

13) AMED-CREST, AMED, Tokyo 100-0004, Japan.
14) Division of Stem Cell Pathology, Center for Experimental Medicine and Systems Biology, Institute of Medical Science, The University of Tokyo, Tokyo 108-8639, Japan.
15) Department of Immunology, Faculty of Life Sciences, Kumamoto University, Kumamoto 860-8556, Japan.

Description of Supplementary information

Supplementary information includes 19 Figures, 4 Tables, and 3 Datasets.

Supplementary Fig. 1

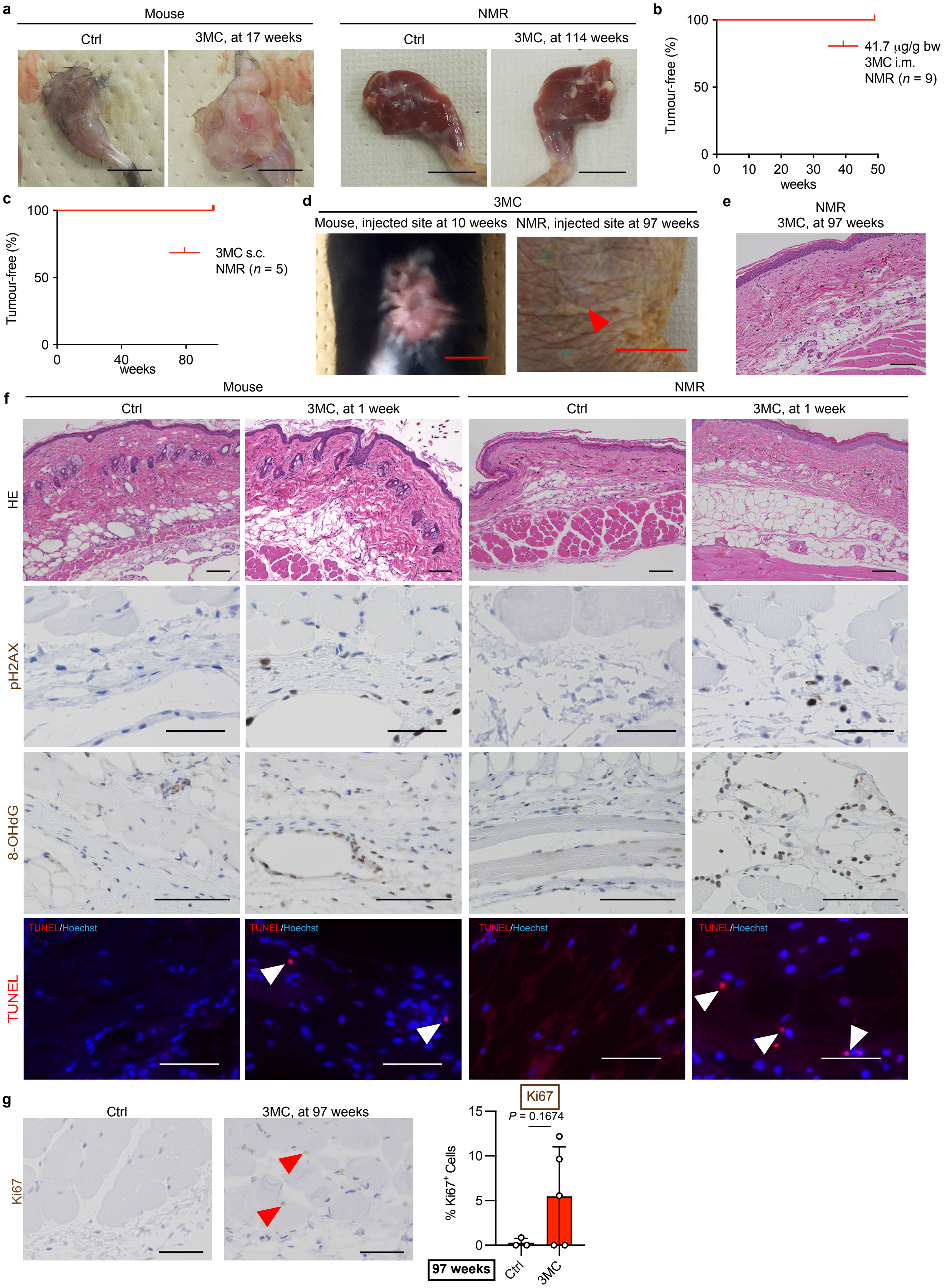

Supplementary Fig. 1. Response of mouse and naked mole-rat (NMR) skin to 3-methylcholanthrene (3MC) treatment.
a, Gross appearance of the mouse limbs at 17 weeks and NMR limbs at 114 weeks after intramuscular injection of 1 mg 3MC. The contralateral legs that were not injected with 3MC served as the control (Ctrl). Scale bar: 1 cm. **b**, Kaplan–Meier curves of tumour-free NMRs treated with 41.7 µg per g body weight (bw) of 3MC. *n* = 9 animals. **c**, Kaplan–Meier curves of tumour-free NMRs after subcutaneous (s.c.) injection of 1 mg 3MC. *n* = 5 animals. **d** and **e**, Gross appearance (**d**) and haematoxylin and eosin (HE) staining (**e**) of mouse back skin at 10 weeks and NMR back skin at 97 weeks after s.c. injection of 1 mg 3MC. Red arrowhead indicates injection site. Scale bars: 1 cm (gross) and 100 µm (HE). *n* = 5 animals per species. **f**, HE staining and immunohistochemical staining for phospho-Histone H2A.X (pH2AX)-, 8-hydroxy-2′-deoxyguanosine (8-OHdG)-, and TUNEL staining of the skin of mice and NMRs at 1 week after 3MC s.c. injection. Scale bars: 100 µm (HE and 8-OHdG), 50 µm (pH2AX and TUNEL). White arrowheads indicate positive cells. **g**, Immunohistochemical staining and quantification of Ki67-positive cells in the skin of NMRs at 97 weeks after s.c. injection of 1 mg 3MC. Red arrowheads indicate positive cells. For quantification, data are presented as the mean ± SD of *n* = 3 (for control) or *n* = 5 (for 3MC) animals. Unpaired *t*-test versus untreated control (Ctrl).

Supplementary Fig. 2

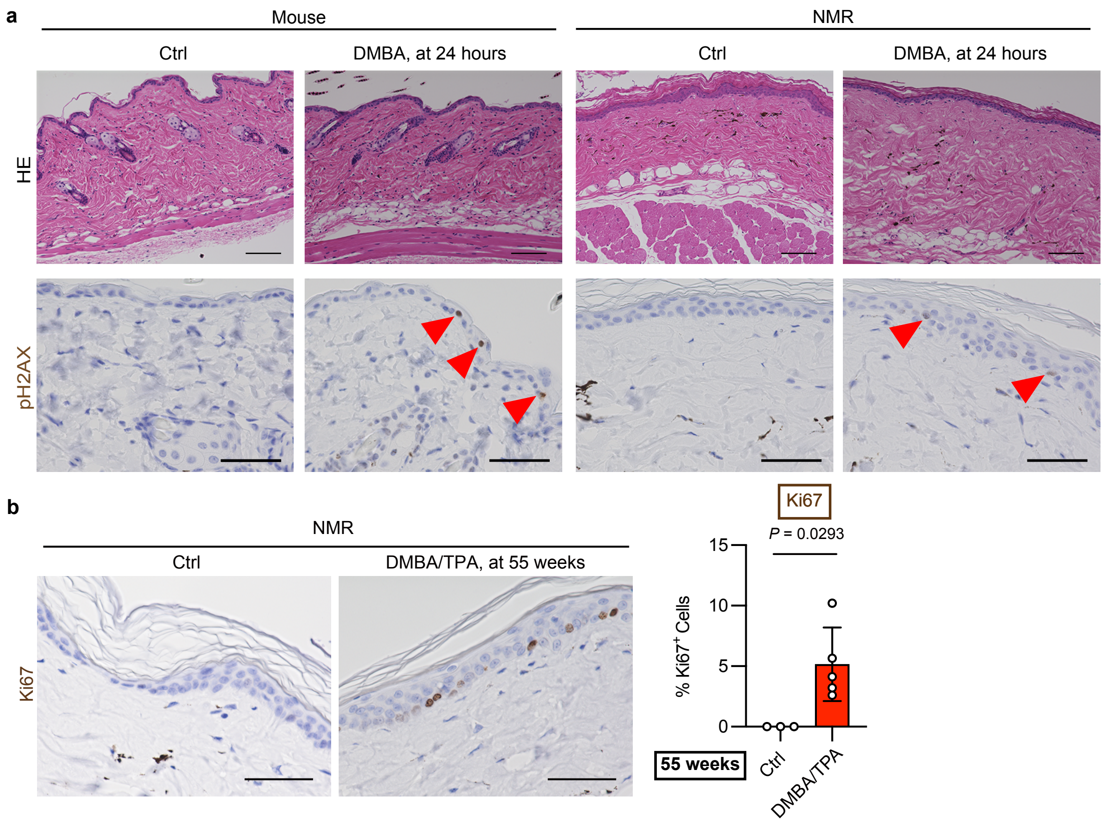

Supplementary Fig. 2. Responses of mouse and naked mole-rat (NMR) skin to 7,12-dimethylbenz[a]anthracene (DMBA)/12-*O*-tetradecanoylphorbol-13-acetate (TPA) treatment.

a, Haematoxylin and eosin (HE) staining and immunohistochemical staining of phospho-Histone H2A.X (pH2AX)-staining in the skin of mice and NMRs at 24 h after DMBA treatment. Scale bars: 100 µm (HE) and 50 µm (pH2AX). Red arrowheads indicate positive cells. **b**, Immunohistochemical staining and quantification of Ki67-positive cells in NMR skin at 55 weeks after starting DMBA/TPA treatment. For quantification, data are presented as the mean ± SD of $n = 3$ (for control) or $n = 5$ (for DMBA/TPA) animals. Scale bar: 50 µm. Unpaired *t*-test versus untreated control (Ctrl).

Supplementary Fig. 3

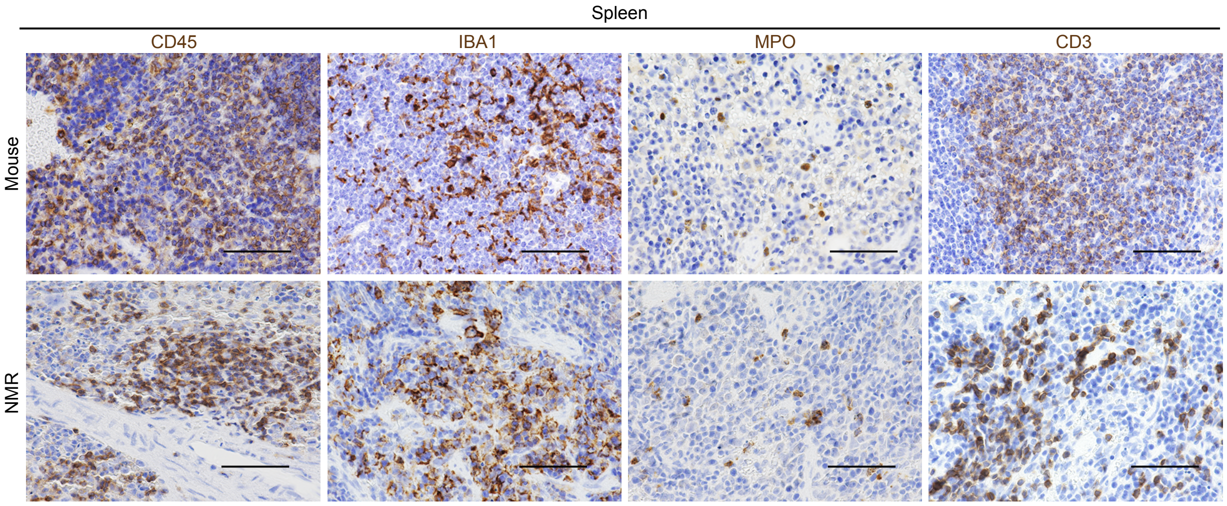

Supplementary Fig. 3. Validation of cell markers by immunohistochemical staining in the mouse and naked mole-rat (NMR) spleen. Immunohistochemical staining for CD45, IBA1, myeloperoxidase (MPO), and CD3 in mouse and NMR spleens. Scale bar: 50 µm.

Supplementary Fig. 4

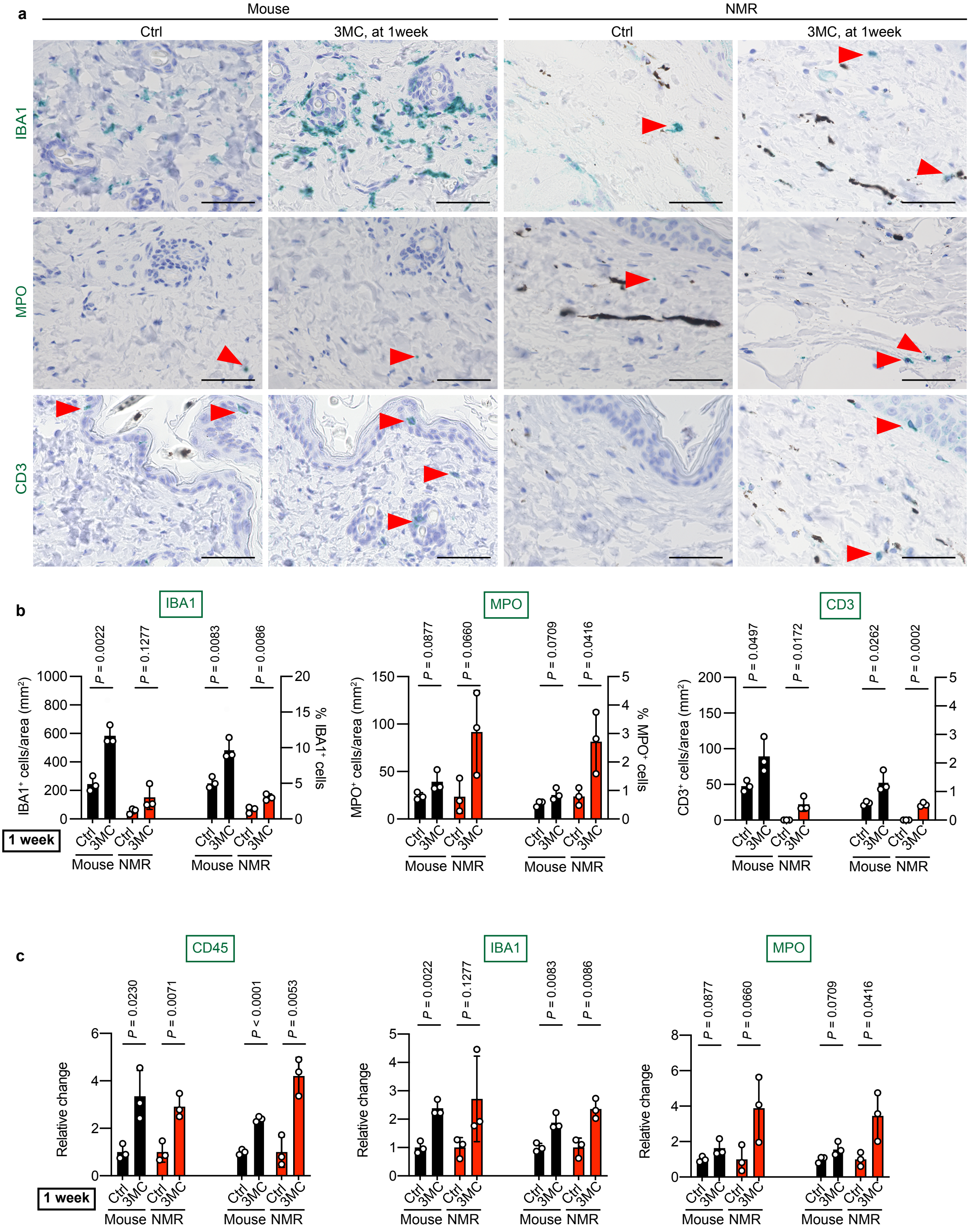

Supplementary Fig. 4. Responses of mouse and naked mole-rat (NMR) skin to 3-methylcholanthrene (3MC) treatment at 1 week.
a, Immunohistochemical detection of IBA1-, MPO-, and CD3-positive cells in the skin of mice and NMRs at 1 week after 3MC subcutaneous (s.c.) injection. Scale bar: 50 µm. Red arrowheads show positive cells. **b**, Quantification of IBA1-, MPO-, and CD3-positive cells per area and total cells in skin sections at 1 week after s.c. injection of 3MC. It is of note that the number of only MPO-positive cells at 1 week after 3MC treatment was higher in NMR skin than in mice. **c**, Relative changes in the number of CD45-, IBA1-, and MPO-positive cells in skin sections at 1 week after s.c. injection of 3MC. The values of Fig. 3c and Supplementary Fig. 3b were recalculated. The values shown are the average fold change relative to that of the untreated control (Ctrl). Relative changes of CD3-positive cells were not calculated because the number of CD3-positive cells in the NMR control was 0. For quantification in **b** and **c**, data are presented as the mean ± SD of *n* = 3 animals per species. Unpaired *t*-test versus untreated control.

Supplementary Fig. 5

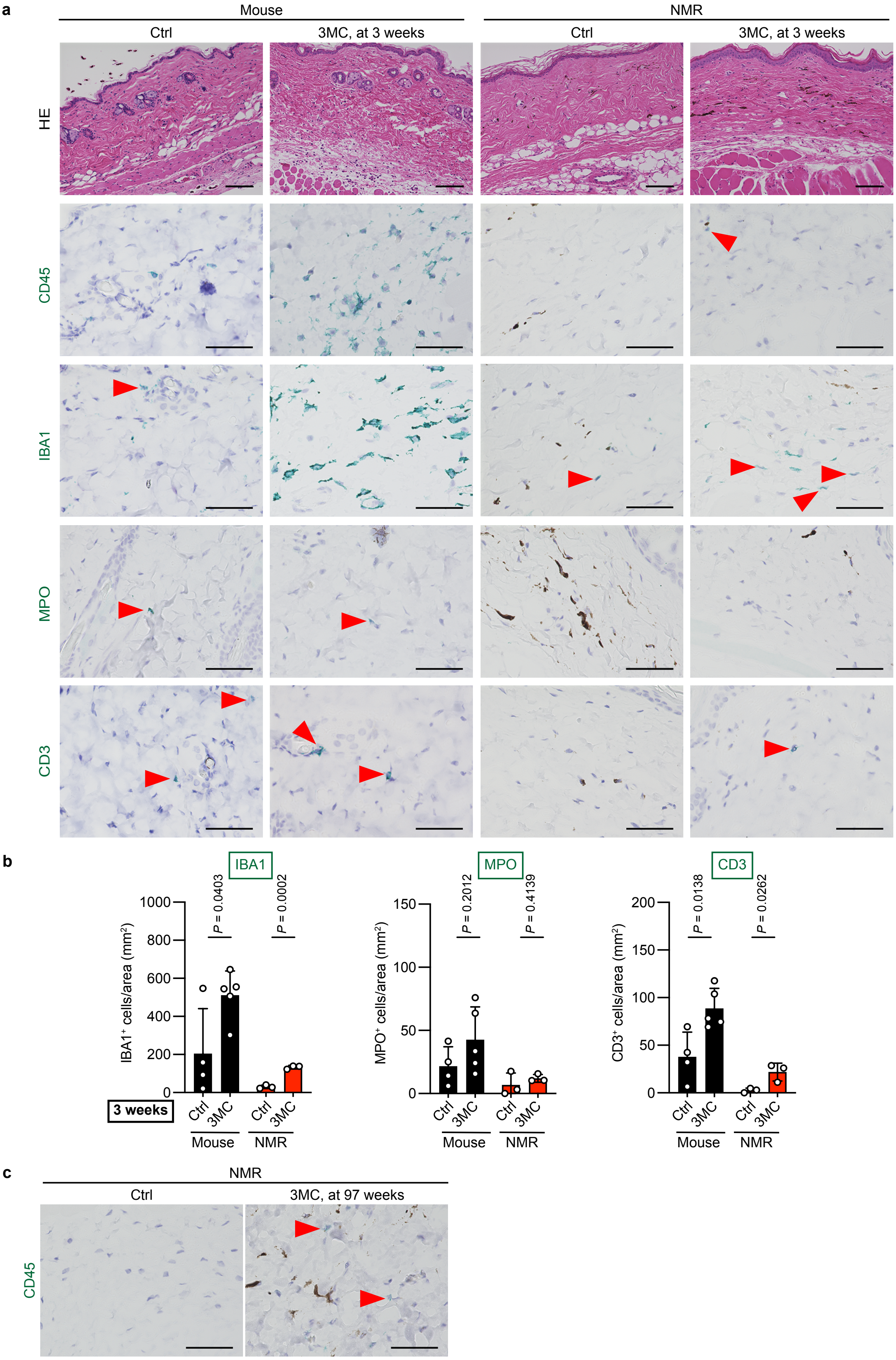

Supplementary Fig. 5. Responses of mouse and naked mole-rat (NMR) skin to 3-methylcholanthrene (3MC) treatment at 3 and 97 weeks. **a**, Haematoxylin and eosin (HE) staining and immunohistochemical detection of CD45-, IBA1-, MPO-, and CD3-positive cells in the skin of mice and NMRs at 3 weeks after 3MC subcutaneous (s.c.) injection. Scale bars: 100 µm (HE) and 50 µm (others). Red arrowheads show positive cells. **b,** Quantification of IBA1-, MPO-, and CD3-positive cells per area in skin sections at 3 weeks after s.c. injection of 3MC. For quantification, data are presented as the mean ± SD of $n = 3$ (for NMR), $n = 4$ (for control mouse), or $n = 5$ (for 3MC mouse) animals. Unpaired t-test versus untreated control (Ctrl). **c**, Immunohistochemical detection of CD45-positive cells in NMR skin at 97 weeks after 3MC s.c. injection. Scale bar: 50 µm. Red arrowheads show positive cells.

Supplementary Fig. 6

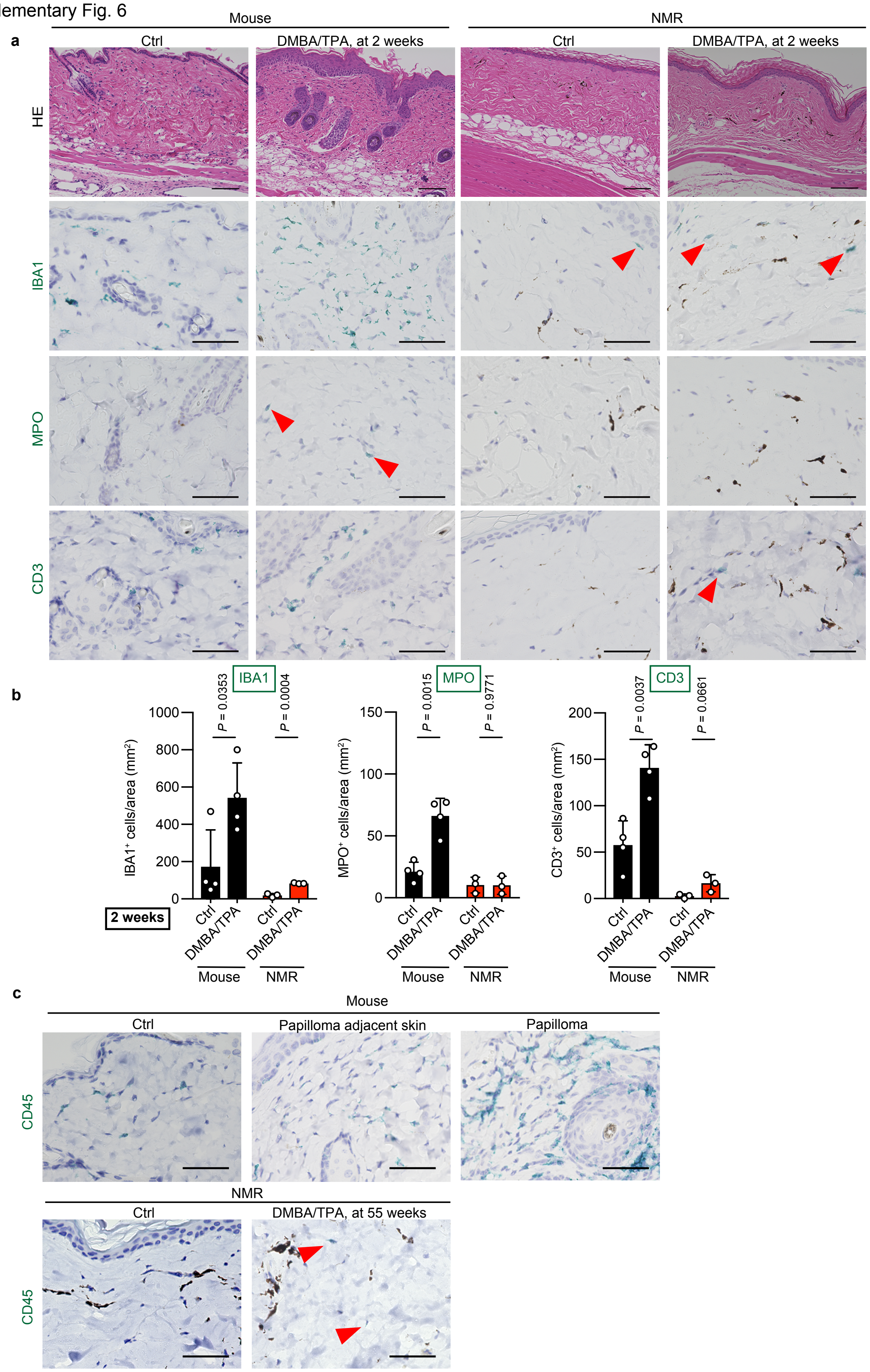

Supplementary Fig. 6. Responses of mouse and naked mole-rat (NMR) skin to 7,12-dimethylbenz[a]anthracene (DMBA)/12-*O*-tetradecanoylphorbol-13-acetate (TPA) treatment at 2 weeks.

a, Haematoxylin and eosin (HE) staining and immunohistochemical detection of IBA1-, MPO-, and CD3-positive cells in the skin of mice and NMRs at 2 weeks after exposure to DMBA/TPA. Scale bars: 100 µm (HE) and 50 µm (others). Red arrowheads show positive cells. **b,** Quantification of IBA1-, MPO-, and CD3-positive cells per area in skin sections at 2 weeks after exposure to DMBA/TPA. For quantification, data are presented as the mean ± SD of $n = 3$ (for NMR) or $n = 4$ (for mouse) animals. Unpaired *t*-test versus untreated control (Ctrl). **c**, Immunohistochemical detection of CD45-positive cells in mouse skin sections at the end point and in NMRs at 55 weeks after exposure to DMBA/TPA. Scale bar: 50 µm. Red arrowheads show positive cells.

Supplementary Fig. 7

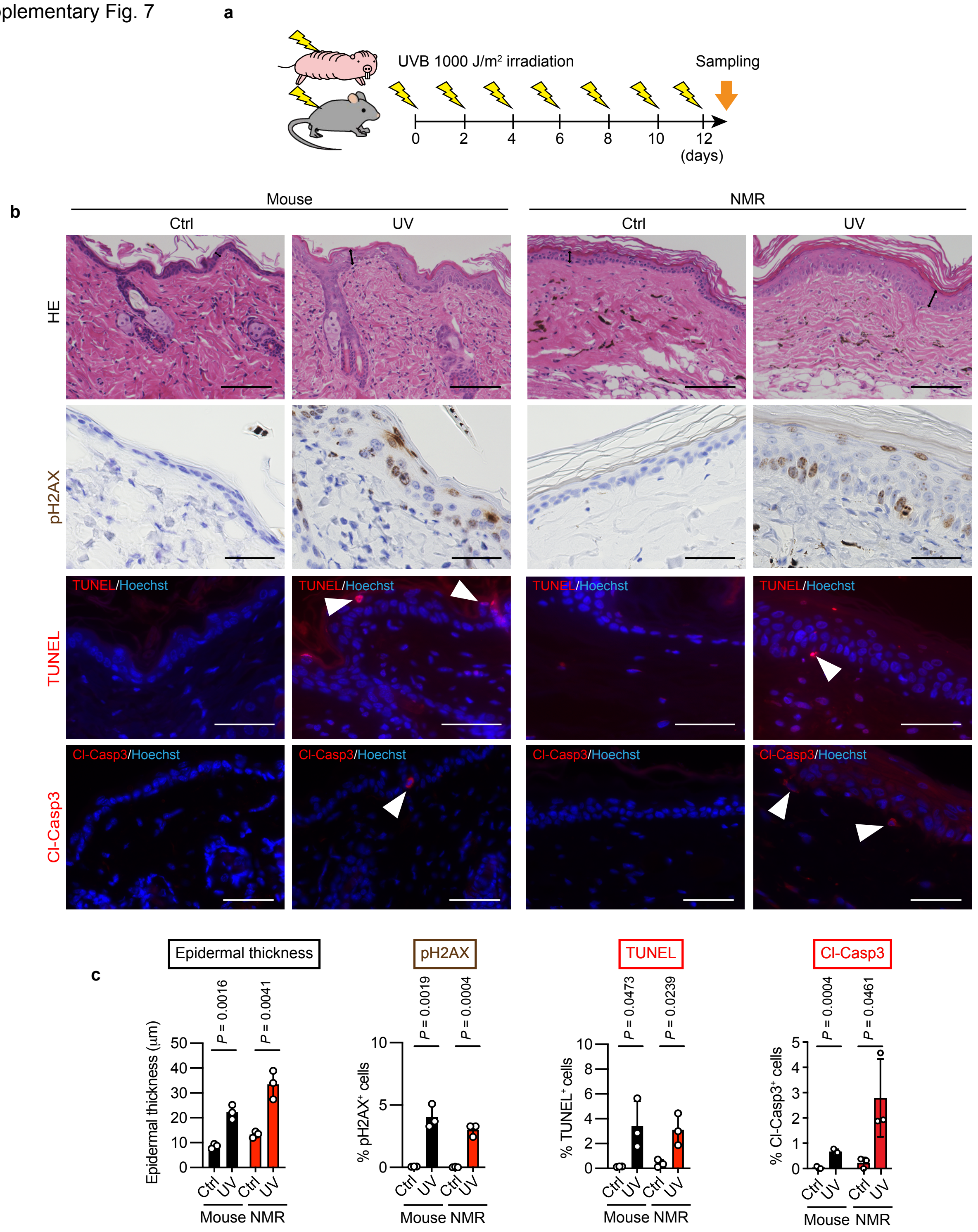

Supplementary Fig. 7. Responses of mouse and naked mole-rat (NMR) skin to UV irradiation.
a, Schematic diagram for investigating responses to UVB irradiation. **b**, Haematoxylin and eosin (HE) staining and immunohistochemical staining for phospho-Histone H2A.X (pH2AX), TUNEL, and cleaved caspase-3 in the skin of mice and NMRs after UVB irradiation. Scale bars: 100 µm (HE) and 50 µm (others). Double-headed arrows show epidermal thickness and white arrowheads show positive cells. **c**, Quantification of epidermal thickness from HE staining images and pH2AX-, TUNEL- and cleaved caspase-3-positive cells per total cells after UVB irradiation. Data are presented as the mean ± SD of $n = 3$ animals. Unpaired t-test versus untreated control (Ctrl).

Supplementary Fig. 8

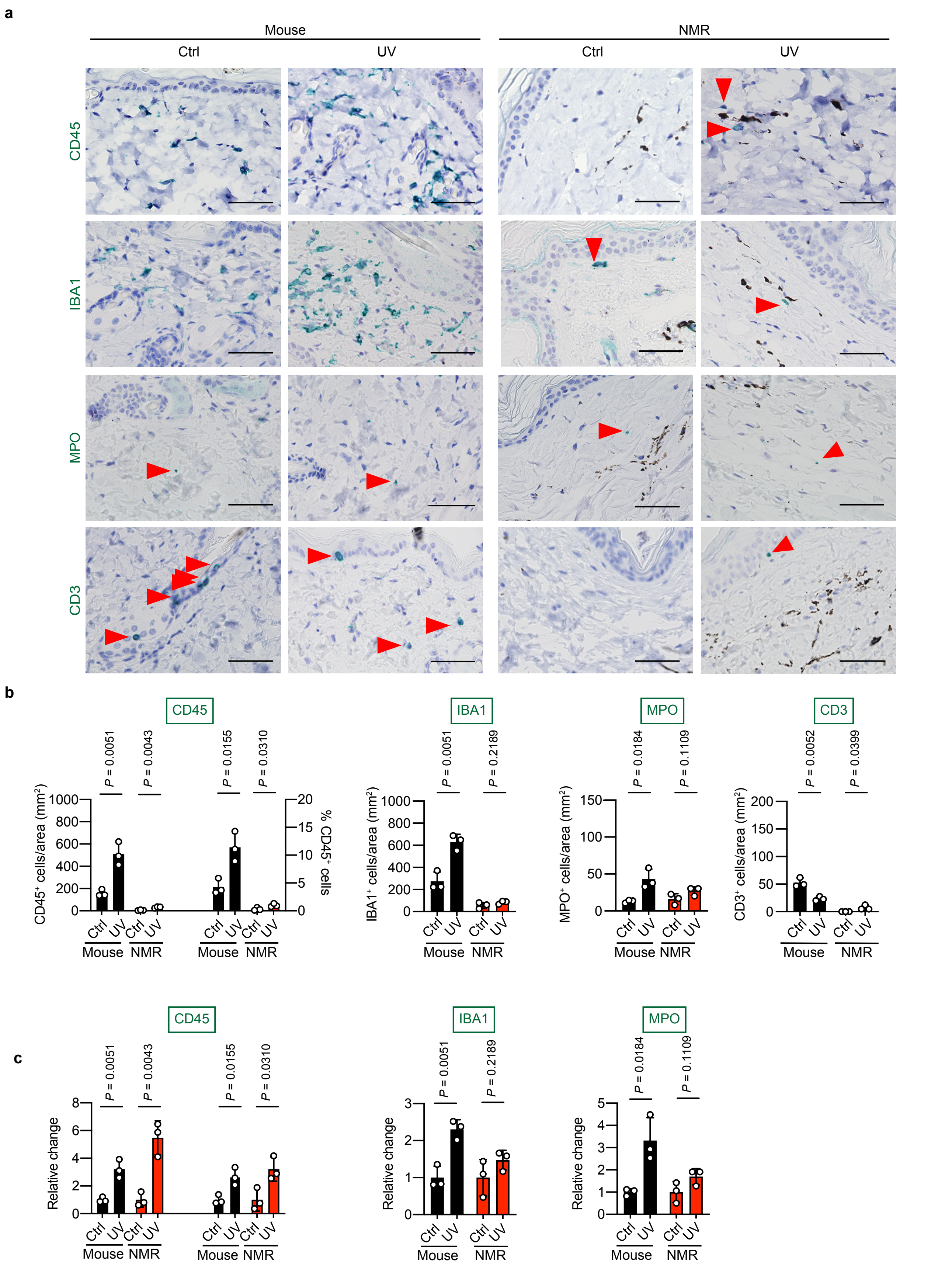

Supplementary Fig. 8. Immune cell infiltration in mouse and naked mole-rat (NMR) skin after UV irradiation.
a, Immunohistochemical staining for CD45, IBA1, MPO, and CD3 in the skin after UVB irradiation. Scale bar: 50 μm. Red arrowheads show positive cells. **b**, Quantification of CD45-, IBA1-, MPO-, and CD3-positive cells per area (and per total cells for CD45) after UVB irradiation. **c**, Relative changes in the number of CD45-, IBA1-, and MPO-positive cells in skin sections after UVB irradiation. The values of Supplementary Fig. 8b were recalculated. The values shown are the average fold change relative to that of the untreated control. Relative change for CD3-positive cells was not calculated because the number of CD3-positive cells in the NMR control was 0. For **b** and **c**, data are presented as the mean ± SD of $n = 3$ animals. Unpaired t-test versus untreated control (Ctrl).

Supplementary Fig. 9

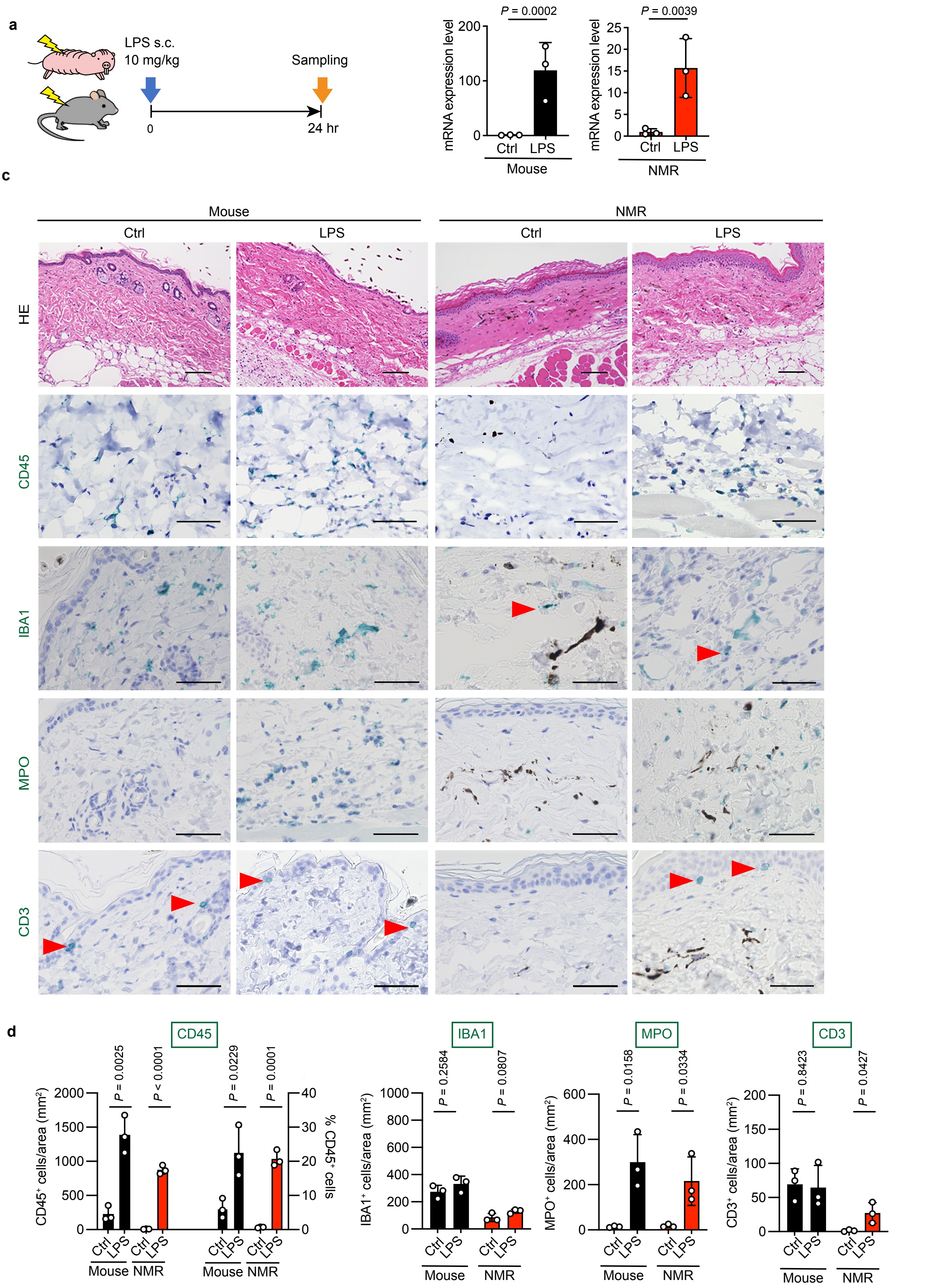

Supplementary Fig. 9. Responses of mouse and naked mole-rat (NMR) skin to lipopolysaccharide (LPS) treatment.
a, Schematic diagram for investigating responses to LPS subcutaneous (s.c.) injection. **b**, Relative interleukin-6 (*IL6*) mRNA levels in the skin of mice and NMRs after LPS injection quantified by RT-qPCR and normalised to actin beta (*ACTB*) mRNA. The values shown are the average fold change relative to that of the untreated control. Primers are listed in Supplementary Table 5. **c**, Haematoxylin and eosin (HE) staining and immunohistochemical staining for CD45, IBA1, MPO, and CD3 in the skin after s.c. injection of LPS. Scale bars: 100 μm (HE) and 50 μm (others). Red arrowheads show positive cells. **d**, Quantification of CD45-, IBA1-, MPO-, and CD3-positive cells per area (and per total cells for CD45) at 24 h after LPS injection. For **b** and **d**, data are presented as the mean ± SD of n = 3 animals. Unpaired t-test versus untreated control (Ctrl).

Supplementary Fig. 10

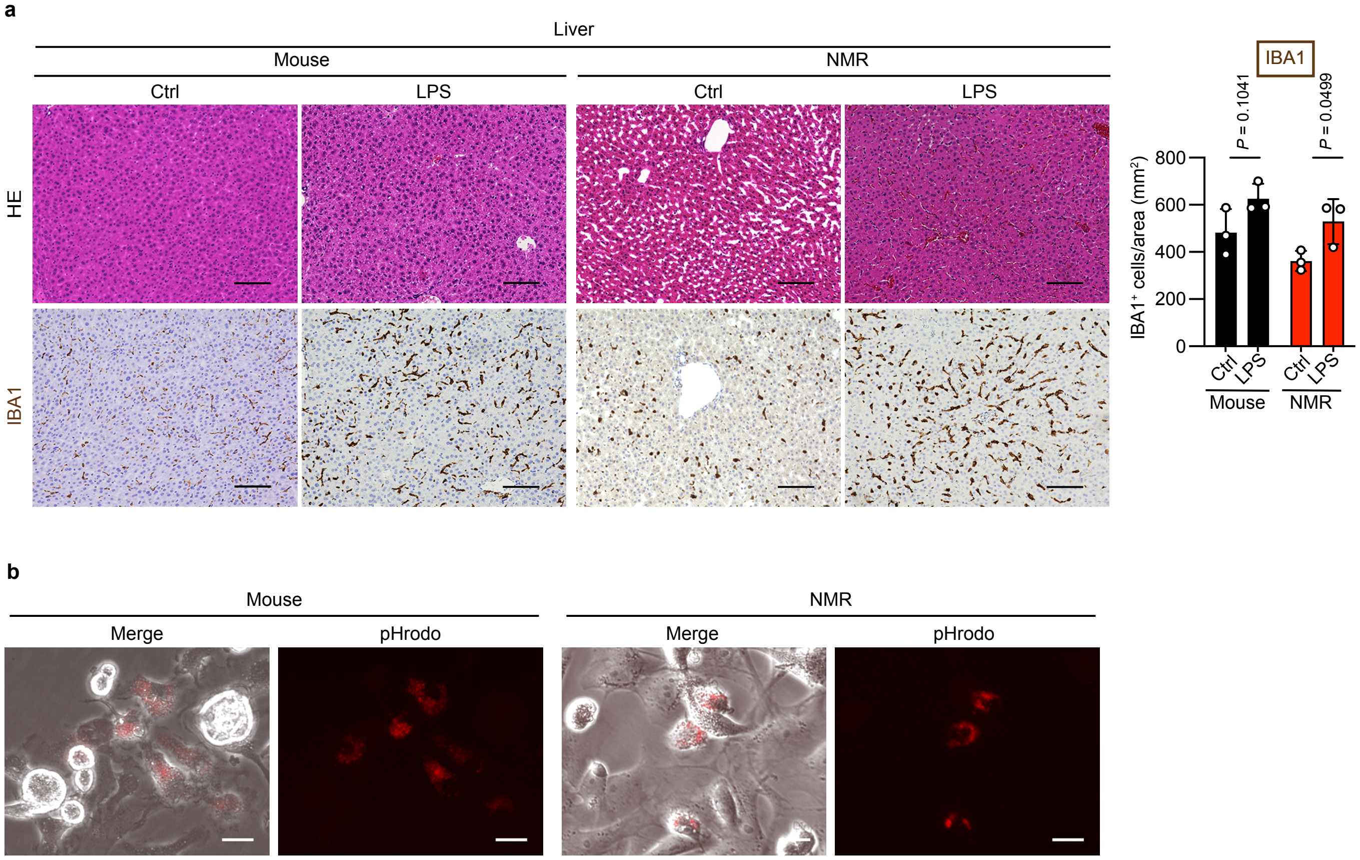

Supplementary Fig. 10. Response of livers to lipopolysaccharide (LPS) and phagocytotic activity of bone marrow-derived macrophages in mice and naked mole-rats (NMRs).

a, Haematoxylin and eosin (HE) staining, immunohistochemical staining, and quantification of IBA1-positive cells in the livers of mice and NMRs at 24 h after intraperitoneal LPS injection. Scale bar: 100 µm. Data are presented as the mean ± SD of n = 3 animals. Unpaired t-test versus untreated control (Ctrl). **b**, Phagocytotic activity analysis of mouse and NMR bone marrow-derived macrophages. Scale bar: 20 µm. Only phagocytosed pHrodo-labelled dead cells show red fluorescence.

Supplementary Fig. 11

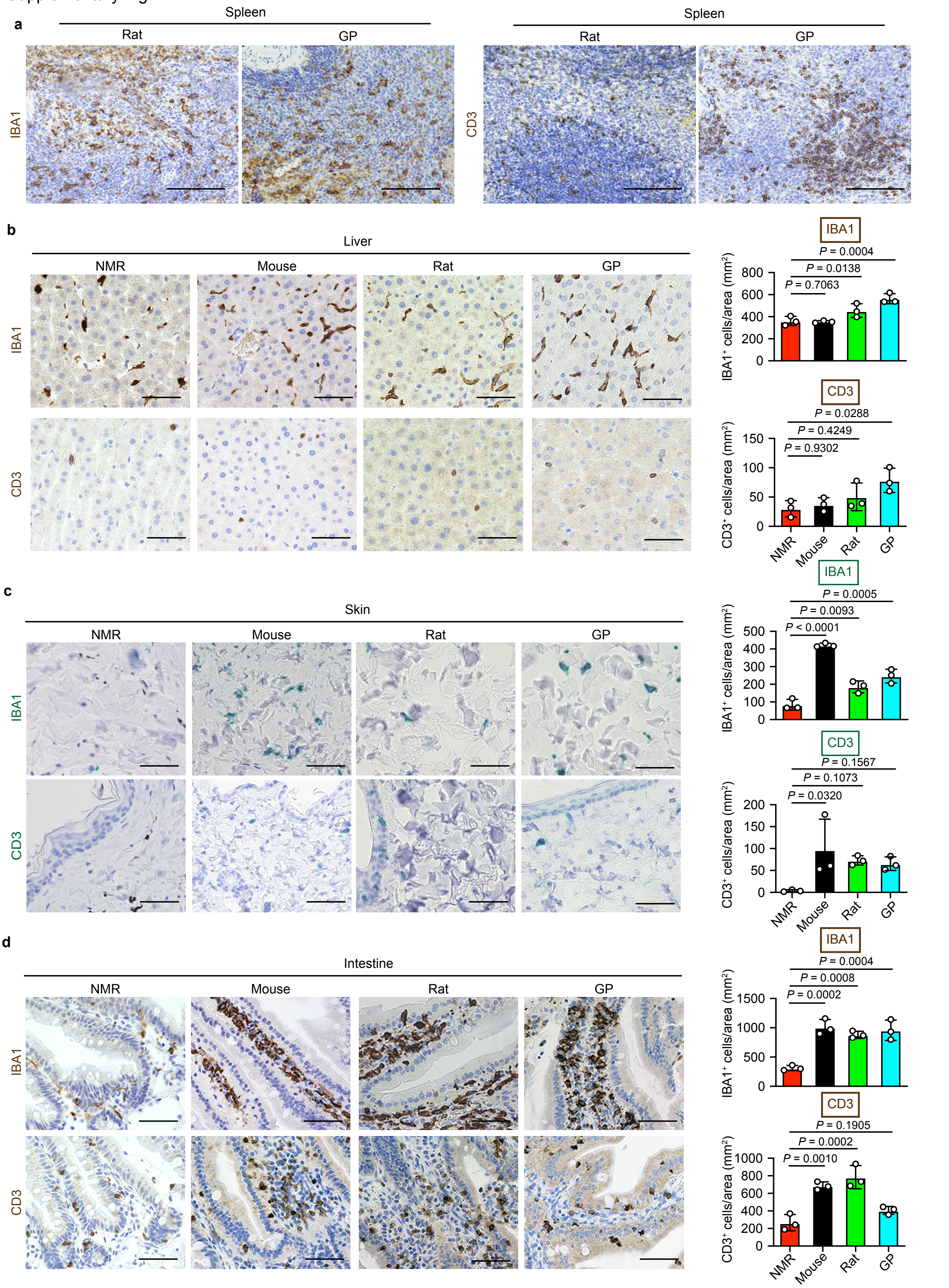

Supplementary Fig. 11. Immunohistochemical analysis of tissue-resident immune cells in several rodent species.
a, Immunohistochemical staining for IBA1 and CD3 in rat and guinea pig (GP) spleens. Scale bar: 100 µm. **b**–**d**, Immunohistochemical staining and quantification of IBA1- and CD3-positive cells per area in liver (**b**), skin (**c**), or small intestine (**d**) sections of naked mole-rats (NMRs), mice, rats, and GPs. Scale bar: 50 µm. Data are presented as the mean ± SD of $n = 3$ animals. One-way ANOVA with Dunnett's multiple comparisons test versus NMR.

Supplementary Fig. 12

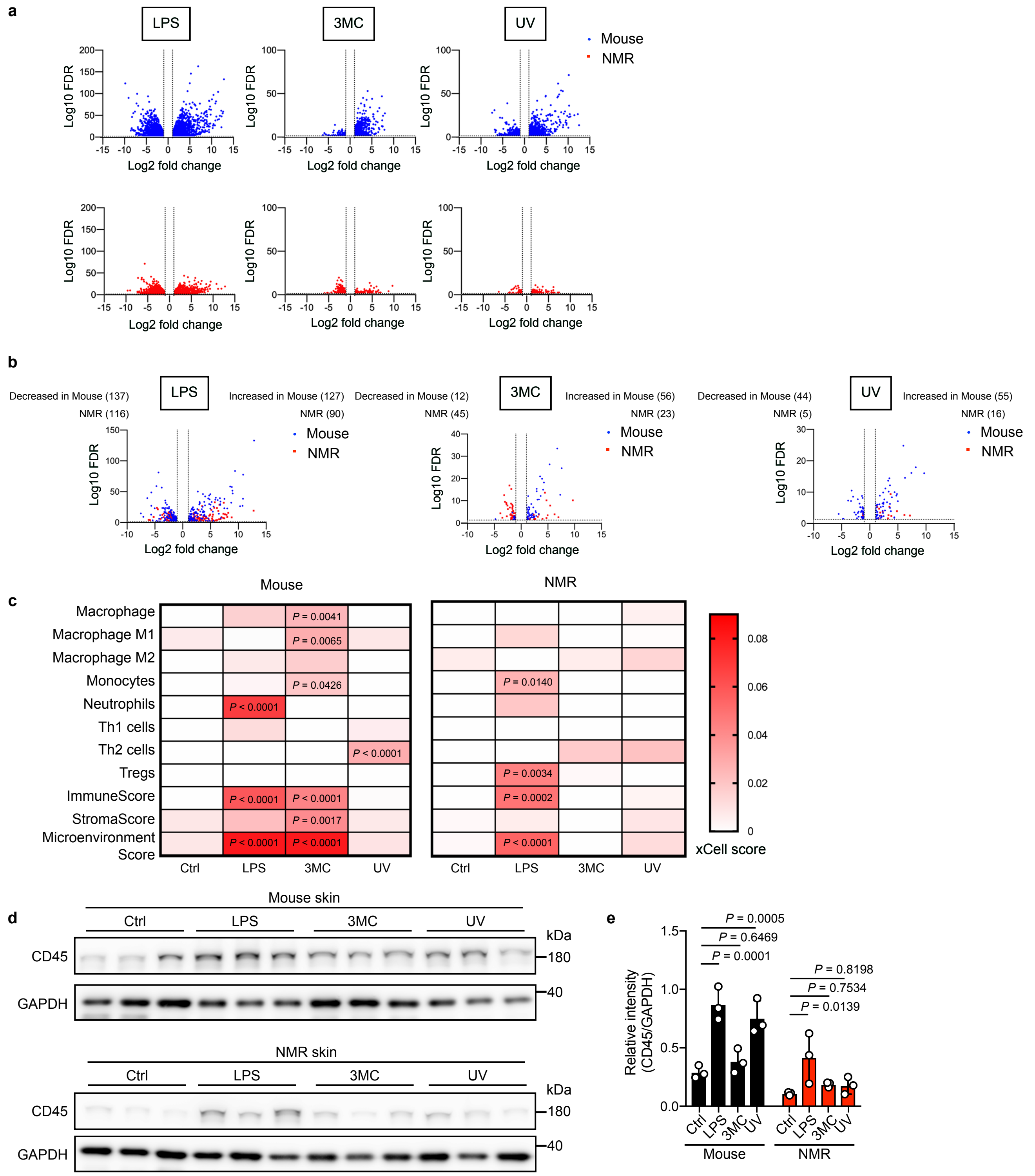

Supplementary Fig. 12. RNA-seq analyses of mouse and naked mole-rat (NMR) skin after exposure to lipopolysaccharide (LPS), 3-methylcholanthrene (3MC), or UV.

a and **b**, Volcano plots of expression differences in all genes (**a**) and 467 selected ligands (**b**, Supplementary Data 1) between mouse skin (blue) and NMR skin (red) after exposure to LPS, 3MC (1 week), or UV. Each point indicates the gene with FDR-adjusted $P < 0.05$ and |log2 fold change| > 1. $n = 3$ animals per treatment. **c**, Heatmap of average scores calculated using xCell. The scores of representative immune cell types are shown. Only significant *P*-values are shown. **d**, Western blot detection of CD45 and GAPDH in the skin of mice and NMRs after LPS, 3MC (1 week), or UV treatment. **e**, Quantification of CD45 expression relative to GAPDH expression. For **c**, data are expressed as the mean of $n = 3$ animals. For **e**, data represent the mean ± SD of $n = 3$ animals. One-way ANOVA with Dunnett's multiple comparisons test versus untreated control (Ctrl).

Supplementary Fig. 13

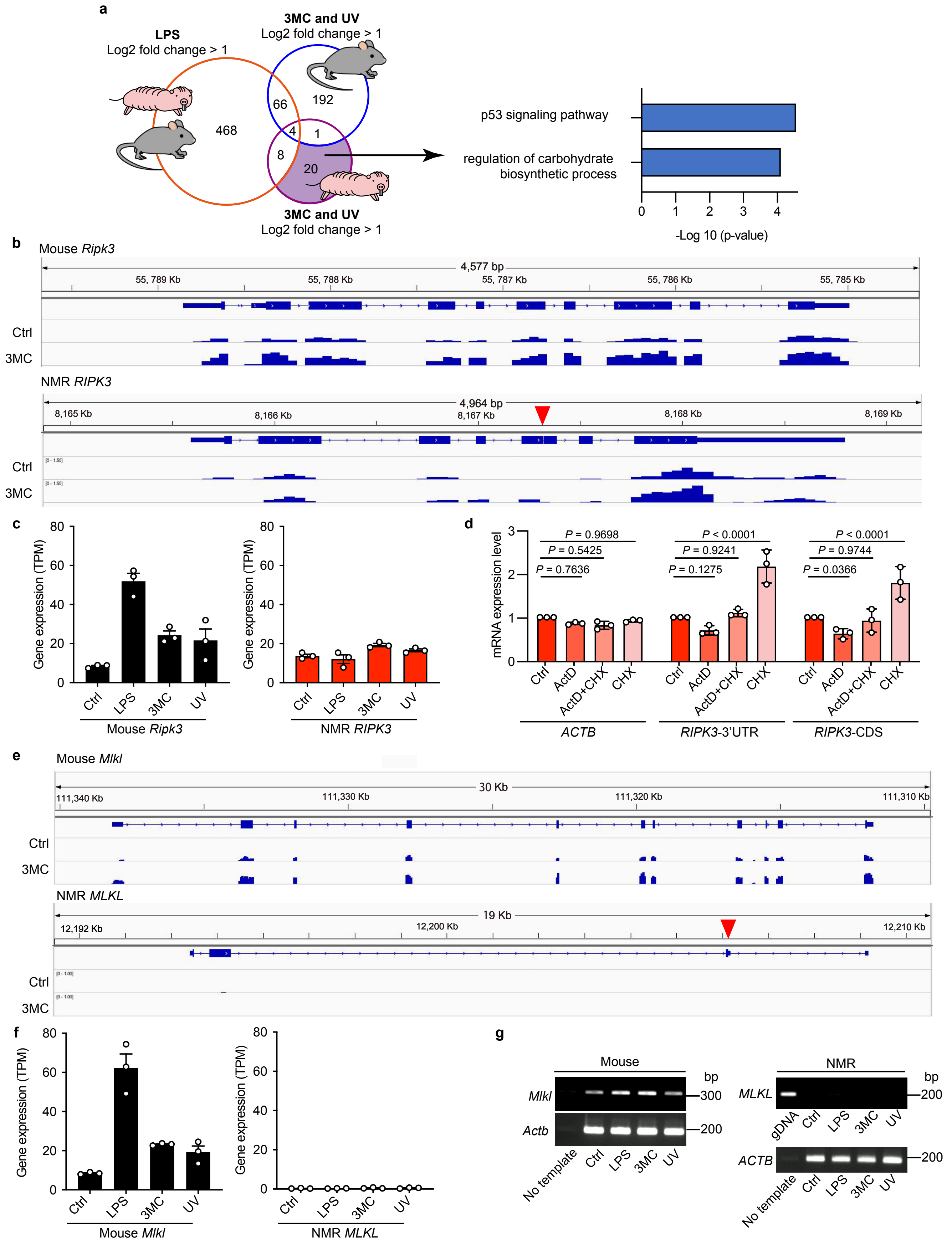

Supplementary Fig. 13. Gene expression analysis of receptor-interacting protein kinase 3 (*RIPK3*) and mixed lineage kinase domain-like (*MLKL*). **a**, RNA-seq analysis. Venn diagram showing the number of commonly upregulated genes in mouse and naked mole-rat (NMR) skin upon lipopolysaccharide (LPS) treatment (orange circle), and upregulated genes in mouse (blue circle) or NMR (purple circle) skin upon both 3-methylcholanthrene (3MC) (1week) and UV treatment; enriched gene ontology (GO) terms and Kyoto Encyclopedia of Genes and Genomes (KEGG) pathways of 3MC-UV NMR-DEGs are shown (purple-filled area, 20 genes). **b**, Visualisation of RNA-seq data at the *RIPK3* gene locus in the control (Ctrl) or 3MC-treated skin, using the Integrative Genomics Viewer (IGV). A red arrowhead indicates a frame-shift mutation in NMR *RIPK3*. **c**, *RIPK3* expression level (transcripts per million, TPM) in Ctrl-, LPS-, 3MC-, or UV-treated skin ($n = 3$ animals in each treatment). **d**, RT-qPCR analysis of NMR fibroblasts incubated with actinomycin D (ActD) and/or cycloheximide (CHX). The values shown are the average fold change relative to that of untreated cells (Ctrl). Data are presented as the mean ± SD of $n = 3$ biological replicates. The mRNA expression was normalised to *GAPDH* expression. Actin beta (*ACTB*) was used as an example of nonsense-mediated mRNA decay non-target mRNA. Two-way ANOVA followed by Dunnett's post hoc test versus untreated control. **e**, Visualisation of RNA-seq data at the *MLKL* gene locus in Ctrl or 3MC-treated skin using IGV. A red arrowhead indicates a frame-shift mutation in NMR *MLKL*. **f**, *MLKL* expression levels (TPM) in Ctrl-, LPS-, 3MC-, or UV-treated skin ($n = 3$ animals per treatment). **g**, Semi-quantitative RT-PCR analysis of the expression of *MLKL* and *ACTB* in skin after each treatment. gDNA: tail-tip genomic DNA used as a PCR positive control.

Supplementary Fig. 14

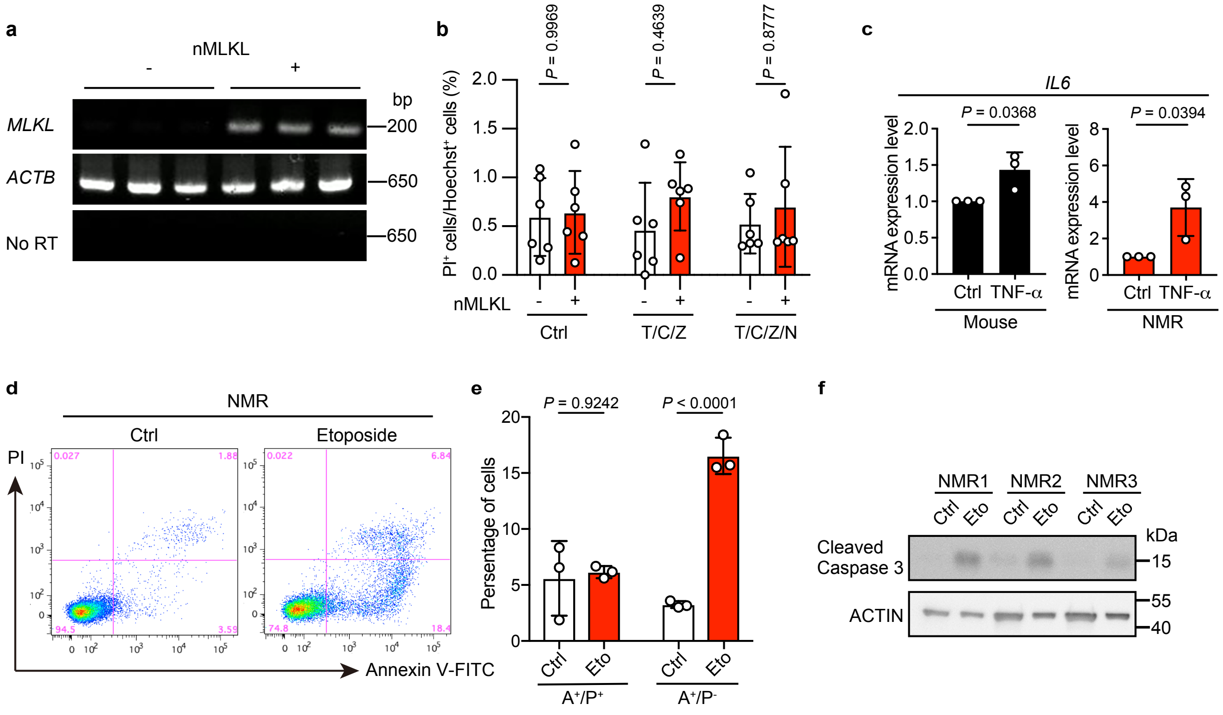

Supplementary Fig. 14. Responses to necroptotic or apoptotic stimuli in skin fibroblasts.
a, Semi-quantitative RT-PCR analysis of the expression of mixed lineage kinase domain-like (*MLKL*) and actin beta (*ACTB*) in naked mole-rat (NMR) SV40ER cells with (+) or without (-) NMR-*MLKL* overexpression. Template without reverse transcriptase (No RT) was used as a negative control. **b**, PI staining of NMR fibroblasts overexpressing NMR-*MLKL*. The cells were treated with a combination of TNF-α (T), cycloheximide (C), z-VAD-fmk (Z), or Nec-1 (N). **c**, Relative expression level of interleukin-6 (*IL6*) mRNA in NMR and mouse fibroblasts treated with TNF-α for 24 h (normalised to *ACTB* mRNA). The values shown are the average fold change relative to that of the untreated control. **d**, Annexin V/PI staining of NMR fibroblasts treated with etoposide. **e**, Quantification of Annexin V- and/or PI-positive cells (%). A^+/P^+, Annexin and PI double-positive cells. A^+/P^-, Annexin single positive cells. Eto: etoposide. **f**, Western blot detection of cleaved caspase-3 and ACTIN in NMR fibroblasts treated with etoposide. $n = 3$ biological replicates. Data are presented as the mean ± SD of $n = 3$ biological replicates (for **c**, **e**) or $n = 6$ independent experiments (for **b**). Unpaired *t*-test versus untreated control (for **c**, **e**) or two-way ANOVA followed by Sidak's multiple comparison test versus control (for **b**).

Supplementary Fig. 15

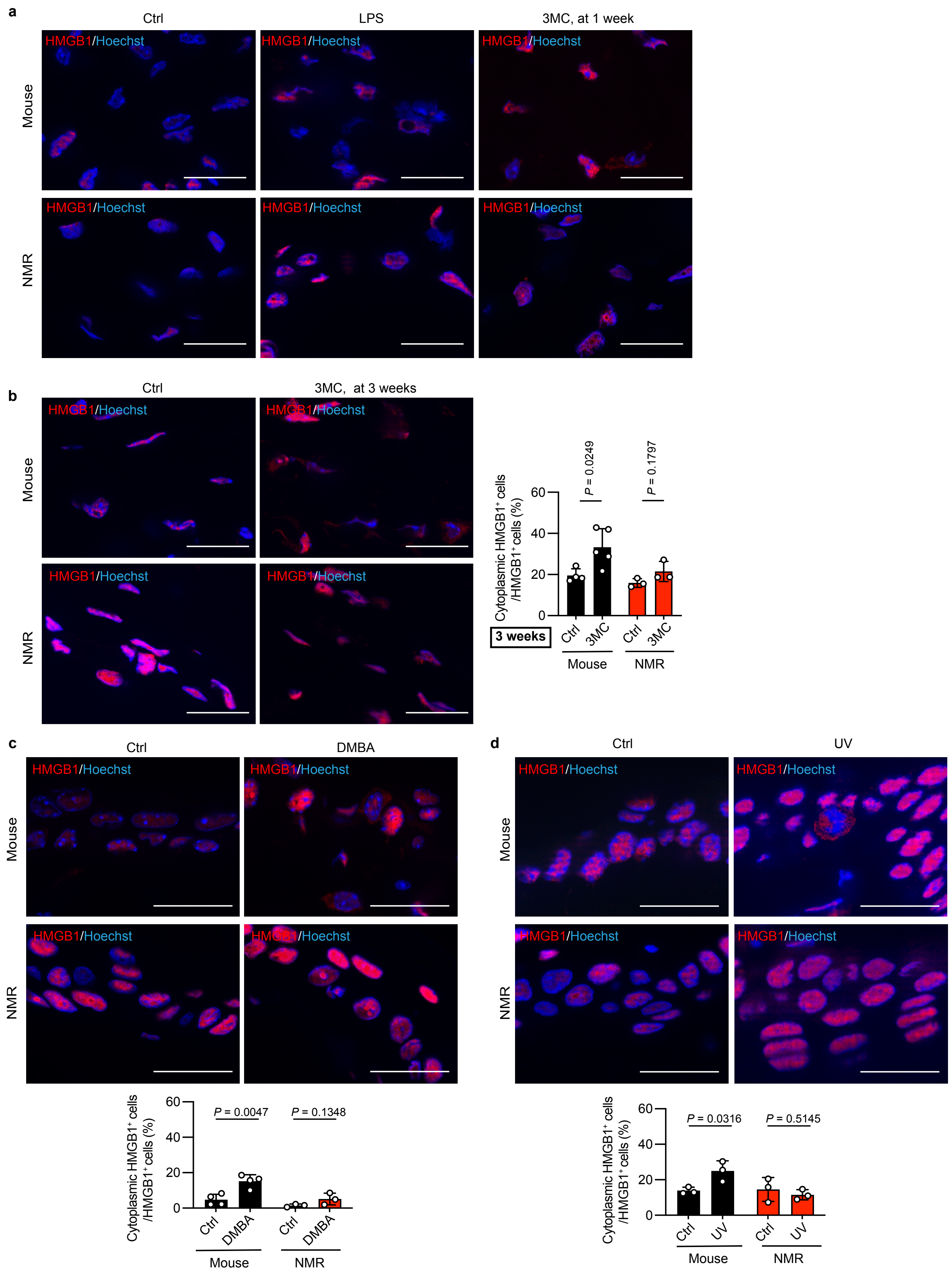

Supplementary Fig. 15. Immunofluorescence staining for high mobility group box-1 protein (HMGB1) in the skin after exposure to lipopolysaccharide (LPS), 3-methylcholanthrene (3MC), 7,12-dimethylbenz[a]anthracene (DMBA), or UV.

a, Immunofluorescence staining for HMGB1 in the skin after exposure to 3MC (1 week) and LPS. Scale bar: 20 µm. **b,** Immunofluorescence staining and quantification of cytoplasmic HMGB1 in the skin at 3 weeks after 3MC treatment. Scale bar: 20 µm. **c,** Immunofluorescence staining and quantification of cytoplasmic HMGB1 in the skin at 24 h after DMBA treatment. Scale bar: 20 µm. **d**, Immunofluorescence staining and quantification of cytoplasmic HMGB1 in the skin after UV treatment. Scale bar: 20 µm. Data are presented as the mean ± SD of n = 3 (for **d** and NMR in **b**, **c**), n = 4 (for **c** and control mouse in **b**), or n = 5 (for 3MC mouse in **b**) animals. Unpaired t-test versus untreated control (Ctrl).

Supplementary Fig. 16

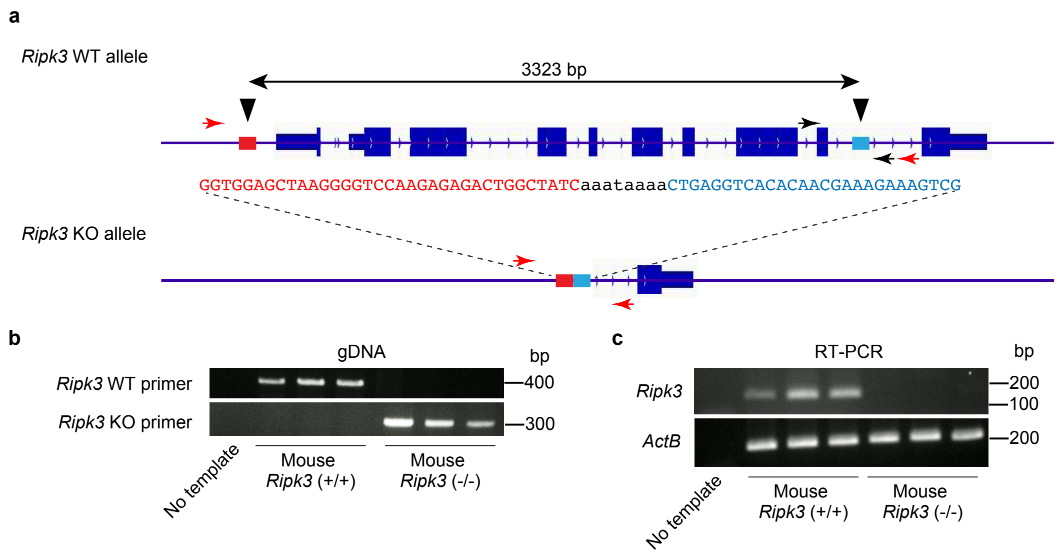

Supplementary Fig. 16. Knockout of mouse receptor-interacting protein kinase 3 (*Ripk3*).
a, Schematic diagram of the *Ripk3* wild-type (WT) allele and the knockout (KO) allele. The targeted alleles of *Ripk3* were generated by introduction of Cas9, the synthetic crRNAs designed to target the 5′ upstream region and intron 9 (arrowheads), tracrRNA, and ssODN into C57BL/6N fertilised eggs. The red and black arrows indicate the primer sets used for detection of KO and WT alleles, respectively. **b**, PCR genotyping of the *Ripk3* alleles. **c**, Semi-quantitative RT-PCR analysis of the skin.

Supplementary Fig. 17

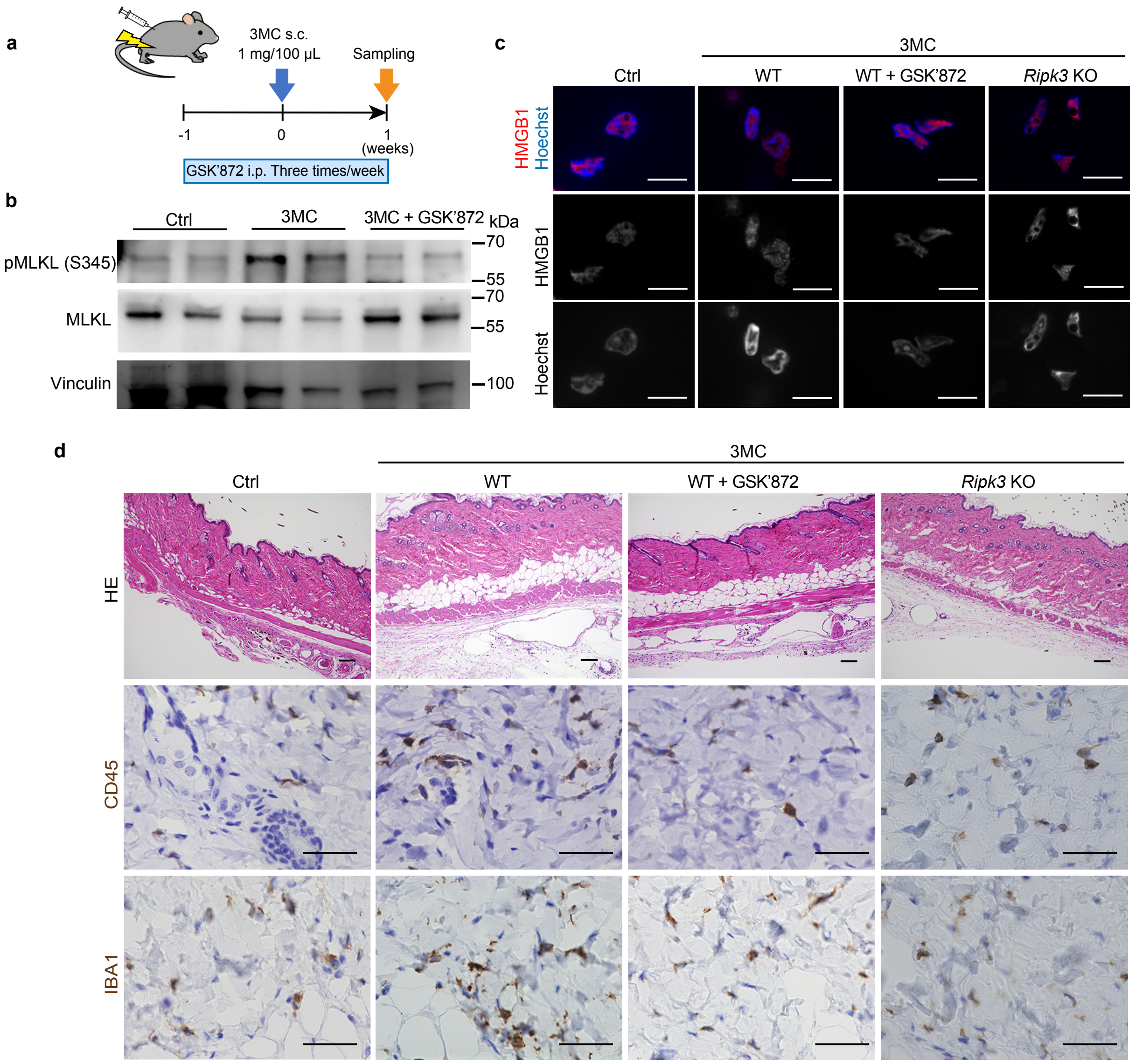

Supplementary Fig. 17. Inhibition of receptor-interacting protein kinase 3 (RIPK3) suppresses necroptosis and inflammatory immune cell responses in mouse skin.

a, Schematic diagram for investigating responses to subcutaneous (s.c.) 3-methylcholanthrene (3MC)-injection after suppression of necroptosis by GSK'872 in mouse skin. GSK'872 was intraperitoneally (i.p.) injected three times a week starting at 1 week before 3MC injection. **b**, Western blot detection of phospho-mixed lineage kinase domain-like (pMLKL [S345]), MLKL, and vinculin in mouse skin after exposure to 3MC with or without GSK'872. *n* = 2 animals per group. **c**, Immunofluorescence staining of cytoplasmic high mobility group box-1 protein (HMGB1) in mouse skin after exposure to 3MC with or without GSK'872. Scale bar: 10 µm. **d**, Haematoxylin and eosin (HE) staining and immunohistochemical staining of CD45- and IBA1-positive cells in mouse skin after exposure to 3MC with or without GSK'872. Scale bars: 100 µm (HE) and 50 µm (others).

Supplementary Fig. 18

Supplementary Fig. 12 NMR CD45

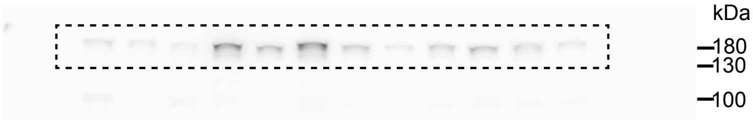

Supplementary Fig. 12 NMR GAPDH

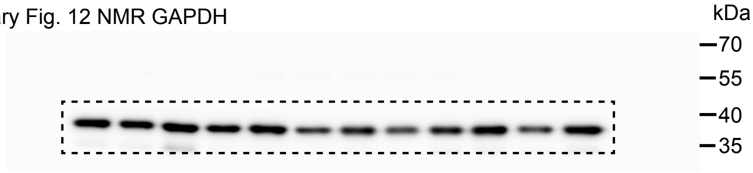

Supplementary Fig. 12 Mouse CD45

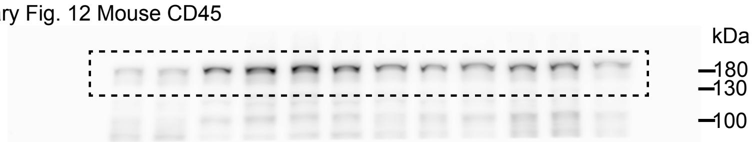

Supplementary Fig. 12 Mouse GAPDH

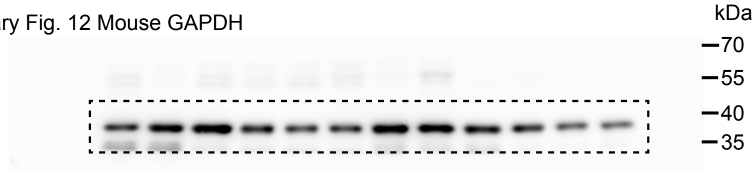

Supplementary Fig. 14 NMR ACTIN

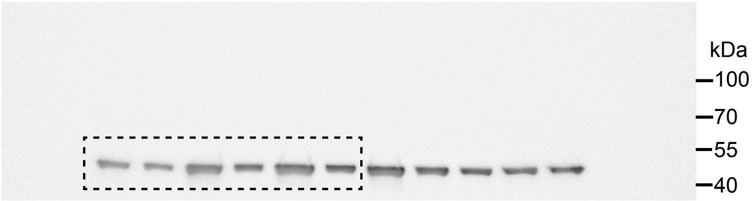

Supplementary Fig. 14 NMR Cleaved Caspase 3

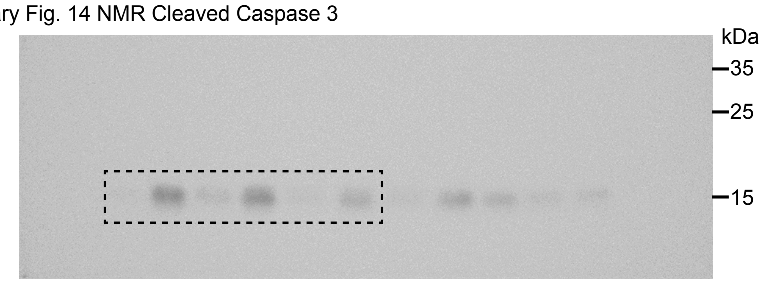

Supplementary Fig. 17 Mouse pMLKL (S345)

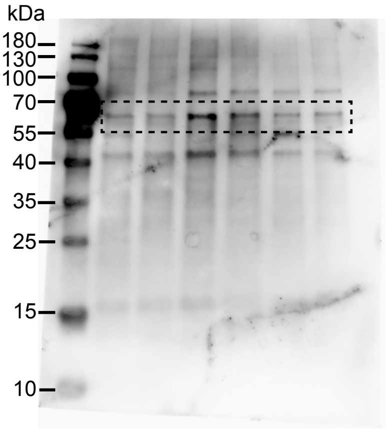

Supplementary Fig. 17 Mouse MLKL

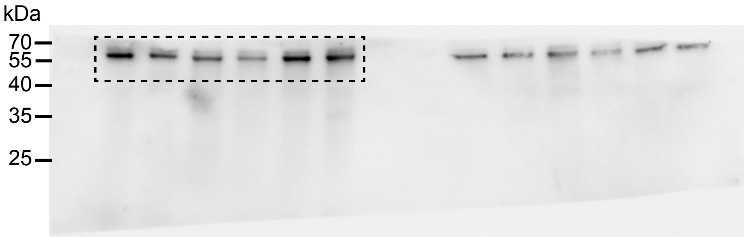

Supplementary Fig. 17 Mouse Vinculin

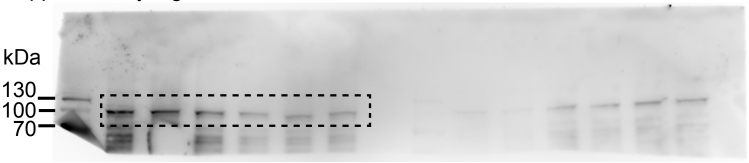

Supplementary Fig. 18. Uncropped versions of blots with size markers.
Uncropped versions of blots related to Supplementary Fig. 12, 14, and 17.

Supplementary Fig. 19

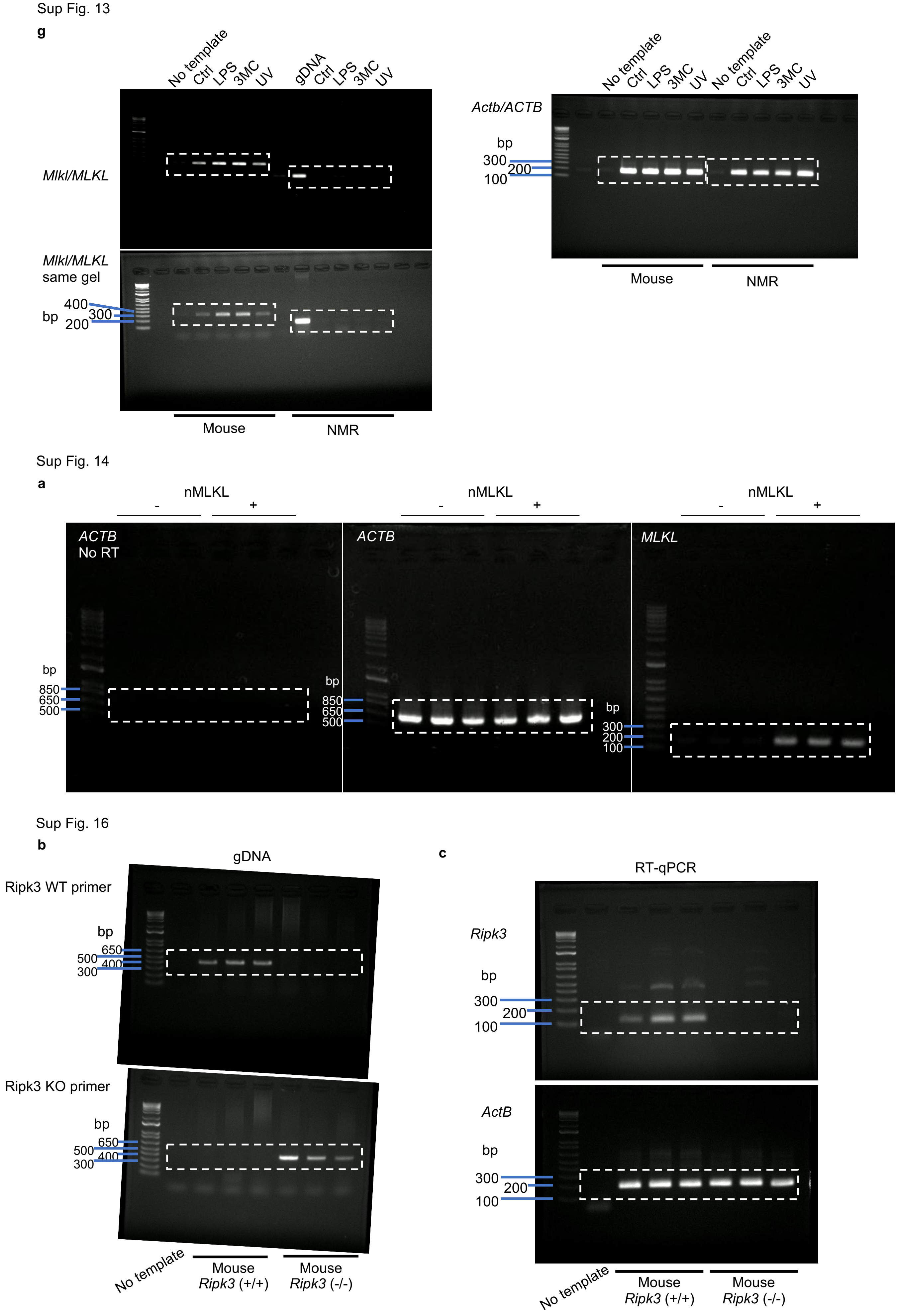

Supplementary Fig. 19. Uncropped versions of gels with size markers. Uncropped versions of gels related to Supplementary Fig. 13, 14, and 16.

Supplementary Table 1: Antibodies used in this study.

Antibody	Code No.	Results	Antigen retrieval and concentration	Epitope	Percent homology (%) to the epitope
CD3 (SP7)	Nichirei (413591)	Positive	Antigen retrieval buffer pH9 (Nichirei), 1:5000	Human CD3, aa 150 to the C-terminus	NMR 86.2%, Mouse 91.4%
Iba1	WAKO (019-19741)	Positive	EDTA pH8 buffer, 1:4000	Rat Iba1, C-terminus	NMR 84.4%, Mouse 93.9%
Myeloperoxidase	DAKO (A0398)	Positive	EDTA pH8 buffer, 1:4000	Human MPO	NMR 87.7%, Mouse 86.4%
CD45	Abcam (ab10558)	Positive	Antigen retrieval buffer pH9 (Nichirei), 1:2000	Human CD45, aa 900-1000	NMR 87.1%, Mouse 91.1%
CD4 (EPR6855)	Abcam (ab133616)	Negative			
CD4 (GHH4)	dianova (DIA-404)	Negative			
CD8 (GHH8)	dianova (DIA-808)	Negative			
CD11b (M1/70)	Biolegend (101201)	Negative			
CD11b (EP1345Y)	Abcam (ab52478)	Negative			
CD34 (EP373Y)	Abcam (ab81289)	Negative			
CD45R(RA3-6B2)	Abcam (ab64100)	Negative			
CD45R(B220)	Pharmingen (01121D)	Negative			
CD68(FA-11)	Abcam (ab64100)	Negative			
CD79a(SP18)	Serotec (MCA1957)	Negative			
EMR1(F4/80)	Original	Negative			
Gr-1	Southern Biotechnology (1900-01)	Negative			
pH2AX	CST (#9718)	Positive	Antigen retrieval buffer pH9 (Nichirei), 1:500	A synthetic phosphopeptide corresponding to residues surrounding Ser139 of human H2A.X	NMR 98.6%, Mouse 97.2%
8-OHdG	Santa Cruz Biotechnology (sc-393871)	Positive	Antigen retrieval buffer pH9 (Nichirei), 1:5000		
Ki67	Abcam (ab16667)	Positive	Antigen retrieval buffer pH9 (Nichirei), 1:200	Human Ki67, aa 1200-1300	NMR 47.5%, Mouse 44.6%
HMGB1	Abcam (ab79823)	Positive	Antigen retrieval buffer pH9 (Nichirei), 1:500	Human HMGB1, aa 150 to the C-terminus	NMR 100.0%, Mouse 97.0%
Cleaved Caspase-3	CST (#9664)	Positive	none, 1:400	Human Caspase-3, amino-terminal residues adjacent to Asp175	NMR 100.0%, Mouse 100.0%
**Antibody**	**Code No.**	**Results**	**Concentration for WB**	**Epitope**	**Percent homology (%) to the epitope**
MLKL	Abcam (ab184718)	Positive	1:1000		
pMLKL	Abcam (ab196436)	Positive	1:1000		
CD45	Abcam (ab10558)	Positive	1:500	Human CD45, aa 900-1000	NMR 87.1%, Mouse 91.1%
Cleaved Caspase-3	CST (#9664)	Positive	1:1000	Human Caspase-3, amino-terminal residues adjacent to Asp175	NMR 100.0%, Mouse 100.0%
β-Actin	CST (#4970)	Positive	1:2000	Human β-actin, near the amino-terminus	NMR 100.0%, Mouse 100.0%
GAPDH	Invitrogen (MA5-15738)	Positive	1:1000	Rabbit GAPDH, Recombinant full length protein	NMR 98.2%, Mouse 94.9%
Vinculin	Sigma-Aldrich (V9131)	Positive	1:1000	Human Vinculin	NMR 99.5%, Mouse 99.1%

Supplementary Table 2: Summary of enriched GO terms and pathways determined by Metascape.

Fig.4a 3MC-UV Mouse-DEGs

GroupID	Category	Term	Description	-LogP	Symbols
1_Summary	GO Biological Processes	GO:0031341	regulation of cell killing	6.066852715	IL12B, IL12RB1, PGLYRP1, RIPK3, CLEC7A, SLAMF6, ITGAL, VCAM1, TNFSF9, TREML2, CCDC88B, SLAMF8, ALOX15, MMP12, FCRLB, SOX15, CD177, GPR55, CTSS, GPR171, IL18BP, BRCA1, IL2RG, NRG1
2_Summary	KEGG Pathway	hsa04060	Cytokine-cytokine receptor interaction	5.624808977	CCR3, IL2RG, IL12B, IL12RB1, TNFRSF9, CCL22, TNFSF9, TNFRSF10B, ALOX15, GPR35, MMP12, VCAM1, IL18BP, BRCA1
3_Summary	GO Biological Processes	GO:0050900	leukocyte migration	5.36324153	BDKRB1, F7, ITGAL, CCL22, VCAM1, TNFRSF10B, RIPK3, SLC7A11, SLAMF8, CD177
4_Summary	Canonical Pathways	M5885	NABA MATRISOME ASSOCIATED	5.354099137	CTSS, F7, F10, NRG1, IL12B, MMP12, PLOD2, CCL22, TNFSF9, INSL6, CLEC7A, P4HA3
5_Summary	Reactome Gene Sets	R-HSA-5213460	RIPK1-mediated regulated necrosis	4.466772045	TNFRSF10B, RIPK3, MLKL, NRG1, IL12B, CEMIP, PSRC1, IL2RG
6_Summary	Reactome Gene Sets	R-HSA-1474244	Extracellular matrix organization	4.304360619	CTSS, ITGAL, MATN1, MMP12, PLOD2, VCAM1, P4HA3, F7, F10
7_Summary	GO Biological Processes	GO:0046579	positive regulation of Ras protein signal transduction	4.280391268	GPR35, NRG1, GPR65, GPR55, BDKRB1, CCR3, CEMIP, TMC8, SLAMF8
8_Summary	Canonical Pathways	M169	PID INTEGRIN2 PATHWAY	3.969876849	F10, ITGAL, VCAM1, IGSF9B, CD177, ROBO3, CDHR1, TREML2, SLAMF6
9_Summary	Reactome Gene Sets	R-HSA-140877	Formation of Fibrin Clot (Clotting Cascade)	3.581700021	F7, F10, CD177, ALOX15, NRG1, MMP12, SOX15, SLC7A11, ITGAL, TNFRSF10B, BDKRB1, KIFC1
10_Summary	GO Biological Processes	GO:0009268	response to pH	3.548836403	CTSS, INSRR, GPR65

Supplementary Fig.13a 3MC-UV NMR-DEGs

GroupID	Category	Term	Description	-LogP	Symbols
1_Summary	KEGG Pathway	hsa04115	p53 signaling pathway	4.554952749	DDB2, MDM2, SESN2, SGK1, CPT1A
2_Summary	GO Biological Processes	GO:0043255	regulation of carbohydrate biosynthetic process	4.112356404	MST1, CD244, SESN2, CPT1A, PGGHG

Supplementary Table 3: Naked mole-rats used in this study.

	ID	Sex	Treatment	Birth	Day of experiments	Use
Chemical carcinogenesis by 3MC muscular injection	L40	M	3MC	16.9.18	17.10.31	Pathological study
	L41	M	3MC	16.9.18	17.10.31	
	L43	M	3MC	16.9.18	17.10.31	
	2B4	M	3MC	17.1.13	18.01.10	Pathological study
	2B7	M	3MC	17.1.13	18.01.10	
	2B9	M	3MC	17.1.13	18.01.10	
	2E11	M	3MC	17.06.16	18.04.29	
	2E14	M	3MC	17.06.16	18.04.29	
	2E21	M	3MC	17.06.16	18.04.29	
Chemical carcinogenesis by 3MC (41.7 µg/g of body weight) muscular injection	L77	F	3MC	19.3.11	21.1.12	
	L78	F	3MC	19.3.11	21.1.12	
	GH3	M	3MC	19.1.10	21.1.12	
	GH4	M	3MC	19.1.10	21.1.12	
	2E25	M	3MC	18.11.2	21.1.12	
	2E26	M	3MC	18.11.2	21.1.12	
	GH24	M	3MC	19.12.11	21.1.13	
	MG23	M	3MC	19.10.11	21.1.13	
	L100	M	3MC	19.8.23	21.1.13	
Chemical carcinogenesis by 3MC subcutaneous injection	2E8	F	3MC	17.06.16	19.03.13	Pathological study
	2E9	F	3MC	17.06.16	19.03.13	Pathological study
	2E12	F	3MC	17.06.16	19.03.13	Pathological study
	C51	M	3MC	18.07.08	19.03.13	Pathological study
	C52	M	3MC	18.07.09	19.03.13	Pathological study
Chemical carcinogensis by DMBA/TPA	2D15	M	DMBA/TPA	18.9.14	19.06.28	Pathological study
	2D17	M	DMBA/TPA	18.9.14	19.06.28	Pathological study
	L66	M	DMBA/TPA	18.9.24	19.06.28	Pathological study
	L67	M	DMBA/TPA	18.9.24	19.06.28	Pathological study
	C54	M	DMBA/TPA	18.7.8	19.06.28	Pathological study
	C58	M	DMBA/TPA	18.7.8	19.06.28	
3MC injection 1 week	Y29	F	3MC 1 wk	17.12.25	18.11.27	Pathologcal study and RNA sequencing
	C53	M	3MC 1 wk	18.7.8	19.04.05	RNA sequencing
	C32	F	3MC 1 wk	16.6.27	18.11.27	Pathologcal study
	2B13	F	3MC 1 wk	17.4.25	18.11.27	Pathologcal study and RNA sequencing
3MC injection 3 weeks	GH28	M	3MC 3 wks	19.12.11	21.1.12	Pathologcal study
	MG21	F	3MC 3 wks	19.10.11	21.1.12	Pathologcal study
	L102	M	3MC 3 wks	19.8.23	21.1.12	Pathologcal study
DMBA treatment 24 hr	MG17	M	DMBA 24 h	19.7.21	21.5.25	Pathologcal study
	BC11	M	DMBA 24 h	20.4.8	21.5.25	Pathologcal study
	2E31	F	DMBA 24 h	18.11.2	21.5.25	Pathologcal study
DMBA/TPA treatment 2 weeks	GH29	M	DMBA/TPA 2 wks	19.12.11	21.1.12	Pathologcal study
	MG24	M	DMBA/TPA 2 wks	19.10.11	21.1.12	Pathologcal study
	L101	F	DMBA/TPA 2 wks	19.8.23	21.1.12	Pathologcal study
LPS subcutaneous injection	C30	M	LPS	16.6.27	19.02.07	Pathologcal study and RNA sequencing
	Y31	N.D.	LPS	17.12.25	19.02.07	Pathologcal study and RNA sequencing
	2B8	F	LPS	17.1.13	19.02.07	Pathologcal study and RNA sequencing
LPS injection (intraperitoneal)	D30	M	LPS	16.8.16	18.09.20	Pathologcal study
	2E16	M	LPS	17.06.16	18.07.30	Pathologcal study
	2B23	F	LPS	17.4.25	18.09.20	Pathologcal study
UV irradiation	Y30	F	UV	17.12.25	19.02.21	Pathologcal study and RNA sequencing
	C46	M	UV	17.1.5	19.01.08	Pathologcal study and RNA sequencing
	2B18	F	UV	17.4.25	19.01.08	Pathologcal study and RNA sequencing
Healthy sample	2B15	F	Control	17.4.25	19.04.12	Pathological study
	C33	F	Control	16.6.27	17.08.04	Pathological study
	C34	M	Control	16.6.27	17.08.10	Pathological study
	C31	F	Control	16.6.27	19.02.08	Pathological study and RNA sequencing
	2B10	F	Control	17.1.23	19.02.08	Pathological study and RNA sequencing
	Y32	M	Control	19.12.25	19.02.08	Pathological study and RNA sequencing

Supplementary Table 4: Primers used in this study.

Gene/primer name	Primer	Sequcence (5'-3')
Ripk3 (Mouse)	Forward	GAAATGGATTGCCCGAGGGA
	Reverse	GTGCTTGCCTCTCAGGACAT
RIPK3 (NMR_CDS)	Forward	CTCATCCCGAGCACCACTTC
	Reverse	CCGCTTCTTCCCAGGTGACAA
RIPK3 (NMR_3'UTR)	Forward	GCGGCCTGTAGGTGTTGAA
	Reverse	GTCAGTTGGGGCATAGCAGG
Mlkl (Mouse)	Forward	AGGAGGCTAACCAGCAGATAGA
	Reverse	GAGCATTGCTTCAGGGTTTTGT
MLKL (NMR)	Forward	ACCTGGGGACTAACTCTGCT
	Reverse	CCACATTCATGCAAACAGCCC
Il6 (Mouse)	Forward	TCTATACCACTTCACAAGTCGGA
	Reverse	GAATTGCCATTGCACAACTCTTT
IL6 (NMR)	Forward	GCTAGTCCTCCACGATGTCC
	Reverse	TTGCCTTTTCCTCCTCTAGGC
ACTB/Actb	Forward	AGACCTTCAACACCCCAGCCATGT
	Reverse	GGCCAGCCAGGTCCAGACGCAG
ACTB/Actb (for Fig. S14a)	Forward	TCCACACAGAGTACTTGCGCTC
	Reverse	TTCAACACCCCAGCCATGTACG
GAPDH/Gapdh	Forward	CTCCTGCGACTTCAACAGCAA
	Reverse	TACCAGGAAATGAGCTTGACA
Ripk3 KO	Forward	AGCGACACCTTGTGATCTCC
	Reverse	CTGGCCCAAGACAACCCTTA
Ripk3 Wild	Forward	GGAAAAGTCAGCCAATCCCG
	Reverse	GCAAGACTAGAGCACACCCTC
