# Supplementary material for: Resistance to chemical carcinogenesis induction via a dampened inflammatory response in naked mole-rats
Source: Commun Biol. 2022 Mar 30;5:287. doi: 10.1038/s42003-022-03241-y (PMC8967925; doi:10.1038/s42003-022-03241-y)
Supplement: Supplementary file 2 — Description of Additional Supplementary Files [file 42003_2022_3241_MOESM2_ESM.pdf]

# Description of Additional Supplementary Files

**File name:** Supplementary Data 1
**Description:** Changes in expression of cell-to-cell communication genes after each stimulation.

**File name:** Supplementary Data 2
**Description:** Summary of enrichment scores determined by xCell.

**File name:** Supplementary Data 3
**Description:** The source data underlying the graphs and charts in the main manuscript.
