# Supplementary material for: Resistance to chemical carcinogenesis induction via a dampened inflammatory response in naked mole-rats
Source: Commun Biol. 2022 Mar 30;5:287. doi: 10.1038/s42003-022-03241-y (PMC8967925; doi:10.1038/s42003-022-03241-y)
Supplement: Supplementary file 6 — Reporting summary [file 42003_2022_3241_MOESM6_ESM.pdf]

nature portfolio

Corresponding author(s): Kyoko Miura

Last updated by author(s): Jan 18, 2022

Reporting Summary

Nature Portfolio wishes to improve the reproducibility of the work that we publish. This form provides structure for consistency and transparency in reporting. For further information on Nature Portfolio policies, see our Editorial Policies and the Editorial Policy Checklist.

Statistics

For all statistical analyses, confirm that the following items are present in the figure legend, table legend, main text, or Methods section.

n/a	Confirmed	
☐	☒	The exact sample size (n) for each experimental group/condition, given as a discrete number and unit of measurement
☐	☒	A statement on whether measurements were taken from distinct samples or whether the same sample was measured repeatedly
☐	☒	The statistical test(s) used AND whether they are one- or two-sided<br>*Only common tests should be described solely by name; describe more complex techniques in the Methods section.*
☒	☐	A description of all covariates tested
☐	☒	A description of any assumptions or corrections, such as tests of normality and adjustment for multiple comparisons
☐	☒	A full description of the statistical parameters including central tendency (e.g. means) or other basic estimates (e.g. regression coefficient) AND variation (e.g. standard deviation) or associated estimates of uncertainty (e.g. confidence intervals)
☐	☒	For null hypothesis testing, the test statistic (e.g. F, t, r) with confidence intervals, effect sizes, degrees of freedom and P value noted<br>*Give P values as exact values whenever suitable.*
☒	☐	For Bayesian analysis, information on the choice of priors and Markov chain Monte Carlo settings
☒	☐	For hierarchical and complex designs, identification of the appropriate level for tests and full reporting of outcomes
☒	☐	Estimates of effect sizes (e.g. Cohen's d, Pearson's r), indicating how they were calculated

Our web collection on statistics for biologists contains articles on many of the points above.

Software and code

Policy information about availability of computer code

Data collection	Microscopic data: All-in-One Fluorescence Microscope (BZ-710; keyence)<br>Immunoblotting data: LAS-4000mini imaging system (Fujifilm)<br>Quantitative real-time PCR: CFX384 Touch Real-Time PCR Detection System (Bio-Rad)<br>RNA sequencing data: NextSeq 550 (Illumina)
Data analysis	Statistical analysis: GraphPad Prism 8<br>Quantitative real-time PCR data: CFX Maestro software (Bio-Rad)<br>Immunofluorescence staining and immunohistochemistry staining analysis: BZ-X700 analyzer software, ImageJ 1.52a (NIH)<br>Immunoblotting analysis: ImageJ 1.52a (NIH)<br>RNA sequencing analysis: cutadapt (ver.1.14), STAR (ver.2.4.1d), HTSeq (version 0.11.2), edgeR (ver. 3.18.1), Metascape, xCell

For manuscripts utilizing custom algorithms or software that are central to the research but not yet described in published literature, software must be made available to editors and reviewers. We strongly encourage code deposition in a community repository (e.g. GitHub). See the Nature Portfolio guidelines for submitting code & software for further information.

Data

Policy information about availability of data

All manuscripts must include a data availability statement. This statement should provide the following information, where applicable:

- Accession codes, unique identifiers, or web links for publicly available datasets
- A description of any restrictions on data availability
- For clinical datasets or third party data, please ensure that the statement adheres to our policy

RNA sequencing data has been deposited in the DDBJ under accession number DRA-010882. The source data underlying Fig. 4a and Supplementary Figs. 12b, c, 13a are provided as Supplementary Tables or Supplementary Datasets. Other data supporting the findings of this study are available from the corresponding author upon reasonable request.

Field-specific reporting

Please select the one below that is the best fit for your research. If you are not sure, read the appropriate sections before making your selection.

☒ Life sciences ☐ Behavioural & social sciences ☐ Ecological, evolutionary & environmental sciences

For a reference copy of the document with all sections, see nature.com/documents/nr-reporting-summary-flat.pdf

Life sciences study design

All studies must disclose on these points even when the disclosure is negative.

Sample size	No statistical method was used to predetermine the sample size. Sample sizes were chosen based on balancing the error in estimating confidence intervals, the cost of replicating experiments, and numbers of naked mole-rats under breeding.
Data exclusions	For histopathological analysis of DMBA/TPA-treatment, one naked mole-rat which had a wound was excluded.
Replication	At least 3 independent experiments were performed to confirm the reproducibility.
Randomization	Samples were allocated into experimental without any intention. Each naked mole-rat was chosen from different colonies.
Blinding	Data collection and analysis were not performed blind. Controls and relative group samples were processed either simultaneously or in parallel in all experiments.

Reporting for specific materials, systems and methods

We require information from authors about some types of materials, experimental systems and methods used in many studies. Here, indicate whether each material, system or method listed is relevant to your study. If you are not sure if a list item applies to your research, read the appropriate section before selecting a response.

Materials & experimental systems

n/a	Involved in the study	
☐	☒	Antibodies
☐	☒	Eukaryotic cell lines
☒	☐	Palaeontology and archaeology
☐	☒	Animals and other organisms
☒	☐	Human research participants
☒	☐	Clinical data
☒	☐	Dual use research of concern

Methods

n/a	Involved in the study	
☒	☐	ChIP-seq
☐	☒	Flow cytometry
☒	☐	MRI-based neuroimaging

Antibodies

Antibodies used	All antibodies for immunostaining for tissues are listed on Supplementary Table 1. anti-CD45 (Abcam, ab10558), MPO (DAKO, A0398), IBA1 (FUJIFILM WAKO, 019-19741), CD3 (Nichirei, 413591), Ki67 (Abcam, ab16667), 8-OHdG (Santa Cruz Biotechnology, sc-393871), pH2AX (Cell Signaling Technology (CST), 9718), HMGB1 (Abcam, ab79823), MLKL (Abcam, ab184718), pMLKL (Abcam, ab196436), Vinculin (Sigma-Aldrich, V9131), Cleaved caspase-3 (CST, 9664), b-Actin (CST, 4970), GAPDH (Invitrogen, MA5-15738), Alexa Fluor 555 anti-rabbit IgG (Thermo Fisher Scientific; A21429), HRP-conjugated anti-rabbit IgG (CST; 7074) and anti-mouse IgG (CST; 7076), HRP-conjugated anti-rabbit, anti-rat and anti-mouse IgG (Nichirei).
Validation	Since no antibodies have been reported for use in immunohistochemistry of naked mole-rat tissues. We validated several commercial antibodies by immunostaining naked mole-rat and mouse spleens by pathologist. The detailed protocol is listed on

Supplementary Table 1. anti-CD45 (Abcam, ab10558) for IHC, MPO (DAKO, A0398) for IHC, IBA1 (FUJIFILM WAKO, 019-19741) for IHC, CD3 (Nichirei, 413591) for IHC, Ki67 (Abcam, ab16667) for IHC, 8-OHdG (Santa Cruz Biotechnology, sc-393871) for IHC, pH2AX (Cell Signalling Technology (CST), 9718) for IHC, HMGB1 (Abcam, ab79823) for IF, MLKL (Abcam, ab184718) for Western blotting (WB), pMLKL (Abcam, ab196436) for WB, Vinculin (Sigma-Aldrich, V9131), b-Actin (CST, 4970) and GAPDH (Invitrogen, MA5-15738)for WB, Cleaved caspase-3 (CST, 9664) for IF, Alexa Fluor 555 anti-rabbit IgG (Thermo Fisher Scientific; A21429) for IF, HRP-conjugated anti-rabbit IgG (CST; 7074) and anti-mouse IgG (CST; 7076), HRP-conjugated anti-rabbit, anti-rat and anti-mouse IgG (Nichirei) for IHC.

Eukaryotic cell lines

Policy information about cell lines

Cell line source(s)	Naked mole-rat skin fibroblasts and macrophages were isolated from 1–2-year-old naked mole-rats. Mouse skin fibroblasts and macrophages were isolated from 6–8-week-old C57BL/6N mice.
Authentication	Cell lines were authenticated based on growth characteristics and unique morphology.
Mycoplasma contamination	We confirmed that naked mole-rat and mouse fibroblasts were mycoplasma-negative by TaKaRa PCR Mycoplasma Detection Set (TAKARA).
Commonly misidentified lines (See ICLAC register)	No commonly misidentified cell lines were used in the study.

Animals and other organisms

Policy information about studies involving animals; ARRIVE guidelines recommended for reporting animal research

Laboratory animals	C57BL/6N mice, Ripk3 KO mice (8–10-week-old) and naked mole-rats (8–30-month-old) were used in this study. All naked mole-rats used for this study were listed on Supplementary Table 3.
Wild animals	No wild animals were used in this study.
Field-collected samples	No field collection of samples was conducted in this study.
Ethics oversight	Animals were maintained in accordance with the regulations designated and approved by the Ethics Committees of Kumamoto University (approval no. A30-043 and A2020-042) and Hokkaido University (approval no. 14-0065), which adheres to the Guide for the Care and Use of Laboratory Animals (United States National Institutes of Health, Bethesda, MD).

Note that full information on the approval of the study protocol must also be provided in the manuscript.

Flow Cytometry

Plots

Confirm that:

☒ The axis labels state the marker and fluorochrome used (e.g. CD4-FITC).

☒ The axis scales are clearly visible. Include numbers along axes only for bottom left plot of group (a 'group' is an analysis of identical markers).

☒ All plots are contour plots with outliers or pseudocolor plots.

☒ A numerical value for number of cells or percentage (with statistics) is provided.

Methodology

Sample preparation	Fibroblasts were dissociated and wash with cold PBS. Then cells were resuspended in 1 x Binding Buffer and stained with Annexin V-FITC and propidium iodide (PI) for 15 min at room temperature in the dark. Cells were mixed with 400 µl of 1 x Binding Buffer and analysed by flow cytometry within 1 h.
Instrument	FACSVerse (BD Biosciences)
Software	FlowJo 10 software (BD Biosciences)
Cell population abundance	More than 15000 cells were counted per sample. No sorting was conducted.
Gating strategy	Analysis was performed with FSC/SSC gating to exclude debris. Gating was determined using unstained control and single-stained controls (Annexin V-FITC and PI) to set up flow cytometer compensation and quadrants.

☐ Tick this box to confirm that a figure exemplifying the gating strategy is provided in the Supplementary Information.
